# Camouflaged membrane-bridged radionuclide/Mn single-atom enzymes target lipid metabolism disruption to evoke antitumor immunity

**DOI:** 10.1186/s40779-025-00647-7

**Published:** 2025-09-19

**Authors:** Meng-Die Yang, Chun-Yan Zhu, Gang Yang, Xiao-Yi Zhang, Yi Zhu, Miao Chen, Jia-Jia Zhang, Ling Bai, Shan-Shan Qin, Chao Ma, Fei Yu, Kun Zhang

**Affiliations:** 1 Department of Nuclear Medicine, Shanghai Tenth People’s Hospital and Institute of Nuclear Medicine, School of Medicine, Tongji University, Shanghai, 200072 China; 2 Central Laboratory, Department of Sichuan Academy of Medical Sciences, Sichuan Provincial People’s Hospital, School of Medicine, University of Electronic Science and Technology of China, Chengdu, 610072 China; 3 Department of Nuclear Medicine, The Central Hospital of Wuhan, Tongji Medical College, Huazhong University of Science and Technology, Wuhan, 430014 China; 4 Department of Pathology, School of Medicine, Duke University, Durham, NC 27710 USA

**Keywords:** Lipid metabolism disruption, Multienzymatical catalysis, Targeted radionuclide therapy (TRT), Single-atom nanozyme-based radiopharmaceuticals, Antitumor immunity

## Abstract

**Background:**

Lipid metabolic reprogramming has been increasingly recognized as a key factor contributing to tumor immune evasion, therapeutic resistance, and plasticity, which collectively compromise the efficacy of targeted radionuclide therapy (TRT). Overcoming the immunosuppressive and hypoxic tumor microenvironment (TME) while interfering with tumor lipid metabolism may offer a promising strategy to potentiate TRT outcomes.

**Methods:**

In this report, a radiopharmaceutical with multienzymatic catalysis activities is developed, wherein tumor cell membrane-coated manganese single-atom nanozymes (Mn/SAE@M) as supports deliver iodine-131 (^131^I) to the tumor. The Mn/SAE nanozyme core was synthesized in situ within hollow mesoporous zeolitic imidazolate frame-8 (ZIF-8) nanoparticles, then coated with homologous tumor cell membranes for targeted delivery and subsequently labeled with ^131^I using the Chloramine-T method. A series of in vitro and in vivo experiments was performed in non-small cell lung cancer (NSCLC) models to evaluate therapeutic efficacy and immune activation.

**Results:**

^131^I-Mn/SAE@M exhibited efficient tumor targeting and internalization mediated by membrane camouflage. Within the TME, the radiopharmaceuticals initiated abundant oxygen (O_2_) release through catalase (CAT)-like catalysis, thereby mitigating a hypoxic microenvironment. In particular, it produced and enriched more reactive oxygen species (ROS) through oxidase (OXD)-, peroxidase (POD)-, and glutathione oxidase (GSHOx)-like catalytic processes. Importantly, ^131^I-Mn/SAE@M activated the cGAS-STING pathway, interfered with the lipid metabolic homeostasis of tumor cells, and induced ferroptosis, which is unraveled to take responsibility for the potentiated antitumor immunity. In bilateral NSCLC tumor-bearing mice, the treatment suppressed both the first and the second tumors, indicating the generation of systemic antitumor immune responses and immunological memory.

**Conclusions:**

Such SAE-based radiopharmaceuticals provide a candidate platform to elevate TRT efficiency, and the proof-of-concept rationale of disrupting lipid metabolic homeostasis through multienzyme-mimicking cascade reactions also provides a general avenue to improve TRT and synergistically magnify antitumor immunity.

**Supplementary Information:**

The online version contains supplementary material available at 10.1186/s40779-025-00647-7.

## Background

Targeted radionuclide therapy (TRT) based on radionuclides including α-particles, β-particles, and Auger electrons has emerged as a promising therapeutic approach across various malignancies [1, 2]. Typically, the β-particles, iodine-131 (^131^I), featuring an 8.03-day half-life, are prevalent for the clinical management of thyroid diseases [3]. Nonetheless, free ^131^I only spontaneously migrated to the thyroid, which means poor accumulation in other tissues or organs, necessitating targeted delivery of ^131^I with nanoparticles [4]. Additionally, the irradiation effectiveness of β-radionuclides is significantly hampered by multiple factors, such as hypoxia and immunosuppressive tumor microenvironment (TME), which are also common limitations of other reactive oxygen species (ROS)-based treatment methods [5–9]. To address it, the combination of TRT with other therapeutic approaches is highly desirable to augment tumor radiosensitivity and attenuate treatment resistance. Developing functional nanovectors to targetly deliver ^131^I and achieve combined TRT with other therapeutic methods holds paramount importance for β-radionuclide-based TRT.

Nanoenzymes, as a type of functional nanomaterial, exert multifaceted actions on different diseases since they could mimic the catalytic efficiency and enzymatic reaction kinetics of natural enzymes [10–12]. As a branch, single-atom nanoenzymes (SAE) resembling those of natural metalloenzymes have been developed [13]. SAE features atomically dispersed catalytically active centers, which, thus, are armed with ultrahigh catalytic abilities that are 10- to 100-fold superior to traditional nanozymes [14, 15]. These characteristics mean that SAE merit to be highlighted as ideal vectors of ^131^I to realize TRT-combined therapy, although this potential has yet to be fully explored. Meanwhile, the cancer cell membrane is usually used to camouflage nanoparticles and endow them with the ability to target homologous cancer cells [16]. Nevertheless, radionuclide labeling on nanoparticles has rarely harnessed the cancer cell membrane as a bridge, let alone ^131^I labeling on SAE.

Given this context, we propose a camouflaged membrane-bridged radiopharmaceutical platform based on ^131^I-labeled Mn SAE (^131^I-Mn/SAE@M), which is designed to produce abundant ROS via multienzyme catalysis and disrupt lipid metabolism, thereby inducing ferroptosis. The subsequent immunogenic effects are anticipated to activate the cGAS-STING pathway and reprogramme TME, eventually enhancing post-TRT antitumor immunity. Specifically, Mn-SAE in-situ grown in hollow mesoporous zeolitic imidazolate frame-8 (ZIF-8) nanoparticles firstly experienced coating with cancer cell membranes and subsequent ^131^I labeling using the Chloramine-T labeling method [4]. Mn-SAE enabled such radiopharmaceuticals to show high targeting specificity and multienzymically catalytic activities, including oxidase (OXD), catalase (CAT), peroxidase (POD), and glutathione oxidase (GSHOx). Through the multienzymatic bio-catalysis, abundant ROS and oxygen (O_2_) release were easily accessible, which united with TRT-based ROS production to enrich ROS content. The excessive ROS is expected to induce glutathione peroxidase 4 (GPX4) down-regulation, trigger ferroptosis, and disrupt the homeostasis of tumor lipid metabolism. Collectively, this study aims to develop and evaluate a promising radiopharmaceutical platform (camouflaged membrane-bridged radionuclide/Mn SAE), which can execute multiple action rationales and signaling pathways to dampen tumor plasticity and boost antitumor immune responses, thereby significantly elevating the therapeutic effects on tumors.

## Methods

### Synthesis of manganese-based single-atom nanozyme (Mn/SAE)

First, zeolitic imidazolate framework-8 hollow nanocubes (ZIF-8 HNCs) were prepared. Zn (NO_3_)_2_·6H_2_O (120 mg) and hexadecyl trimethyl ammonium bromide (CTAB) (4 mg) were dissolved in water (4 ml). Subsequently, an aqueous solution (28 ml) containing 2-methylimidazole (1.816 g) was added to the mixture. The above mixture was stirred vigorously for 2 h at room temperature to produce ZIF-8. The resulting product is centrifuged, washed 3 times with water, and freeze-dried overnight in a vacuum at a low temperature. The obtained ZIF-8 nanocubes (ZIF-8 NCs) (80 mg) were then dispersed in water (12 ml) and ultrasonicated for 30 min at room temperature. After forming a uniform dispersion, 8 ml of aqueous tannic acid (TA, 25 mg/ml) was added to the mixture, and then stirred at room temperature for 2 h. The obtained ZIF-8 HNCs solution was centrifuged to remove the precipitate and placed in a low-temperature vacuum to lyophilize for 6 h. ZIF-8 HNCs powder (160 mg) was ultrasonically dispersed in water (80 ml) at room temperature for 5 min. After forming a homogeneous mixture, MnCl_2_ aqueous solution (1600 μl, 100 mg/ml) was slowly injected into the mixture. Then, the resulting mixture was stirred vigorously at room temperature for 3 h so that the salt solution was fully absorbed. After overnight drying in a vacuum at low temperature, the sample was placed in a tube furnace and heated to 900 °C under argon gas (10 ml/min) for 8 h to obtain Mn/SAE.

### Preparation of cancer cell membrane-coated Mn/SAE (Mn/SAE@M)

In this study, we selected the Lewis lung carcinoma (LLC) cell line, a widely used model derived from non-small cell lung cancer (NSCLC), experimental cell model. LLC cells were grown at 37 °C in a humidified environment with 5% CO_2_ and Dulbecco’s modified Eagle medium (DMEM) containing 10% fetal bovine serum (FBS). After cells in a good growth state were spread over the culture flasks, they were digested and collected by centrifugation, then washed twice with cold phosphate-buffered saline (PBS) Buffer. According to the instructions of the membrane protein extraction kit, lysate was added to the cell samples to break the cells, and the precipitate was obtained by centrifugation at 4 °C and 14,000 rpm for 10 min. An extraction reagent was added to the precipitate, and the supernatant, separated by centrifugation again, contained the cell membrane proteins, which were quantified using the bicinchoninic acid protein assay kit. The extracted LLC cell membranes were mixed with Mn/SAE, sonicated for a while, and then stirred overnight by a magnetic stirrer to coat the LLC cell membranes on the surface of Mn/SAE.

### Radiolabeling and labeling product stability assays

Mn/SAE@M was labeled with ^131^I by the Chloramine T method. To label the nanoparticles, 1 ml of Mn/SAE@M (1 mg/ml) was mixed with Na^131^I (1 mCi) solution. Chloramine T solution (2 μg/μl) was then added, and the mixture was shaken and allowed to react for 5 min. Finally, Na_2_S_2_O_5_ (2 μg/μl) was added to terminate the labeling reaction. The mixed solution was then purified by centrifugation at 4 °C and 14,000 rpm for 10 min, and the precipitate was resuspended with PBS to obtain the purified labeled product. The purified ^131^I-Mn/SAE@M was co-incubated with PBS and FBS (100 μl for each) at 37 °C for 4, 12, 24, and 48 h. To ensure safety during material characterization, radioactive ^131^I was replaced with non-radioactive iodine (KI) and the same conjugation procedure. The energy-dispersive X-ray spectroscopy (EDS) mapping was used to analyze the distribution of iodine. The labeling rate, release profile, and stability of ^131^I-Mn/SAE@M at each time point were determined by paper chromatography and a gamma counter.

### Cell uptake assessment

The solution containing rhodamine B was stirred with ^131^I-Mn/SAE@M for 24 h at room temperature in the dark to synthesize rhodamine B-labeled ^131^I-Mn/SAE@M. At the same time, LLC cells were seeded in confocal dishes (1 × 10^5^ cells per dish). After 24 h of culture, the old medium was discarded and replaced with rhodamine B-labeled ^131^I-Mn/SAE@M (1 ml, 100 μg/ml) and continued to incubate at 37 °C for 1, 2, 4, and 6 h. After reaching the corresponding preset duration, the medium was removed, and the cells were washed twice with PBS buffer. Fluorescence imaging was then observed under a confocal fluorescence microscope.

Furthermore, the extent of Mn/SAE or Mn/SAE@M uptake by LLC cells at 3, 6, 9, 12, and 18 h was quantified by inductively coupled plasma-mass spectrometry (ICP-MS). LLC cells were inoculated in a 10 cm petri dish for 24 h and then added with Mn/SAE or Mn/SAE@M (400 μg for each). At the designated time, the cells were washed 3 times with PBS and then digested with pancreatic enzymes. Then, each group of cells was collected and counted separately. Finally, the elemental Mn content in the cells was detected by ICP-MS.

### Analysis of intracellular ROS generation

The ability of drugs to induce intracellular ROS production was assessed by the fluorescence changes caused by ROS oxidation of 2'-7'-dichlorodihydrofluorescein diacetate (DCFH-DA), dihydroethidium (DHE), hydroxyphenyl fluorescein (HPF), and MitoPY1. LLC cells were grown in 6-well plates (5 × 10^5^ cells per well) for 24 h. Then, cells were treated with ^131^I (500 μCi/ml), Mn/SAE (300 μg/ml), and ^131^I-Mn/SAE@M (500 μCi/ml) for 24 h. The cells were collected, Suspended in diluted DCFH-DA, and then placed in a 37 °C cell culture incubator for staining. The cells were then washed 3 times with serum-free DMEM to adequately remove assays that had not entered the cells. Finally, the fluorescence intensity was analyzed by flow cytometry to quantify the intracellular ROS levels (·O_2_^−^, ·OH, H_2_O_2_). Data were processed on Flow Jo software.

### Animal model construction

The C57BL/6 mice (female, 6 weeks old, *n* = 117) used in the experiment were provided by the animal center of Shanghai Tenth People’s Hospital Clinical Medicine Science and Technology Innovation Park and raised in a specific pathogen-free environment. All animal experiments were reviewed and approved by the Experimental Animal Welfare Ethics Committee of Shanghai Tenth People’s Hospital (SHDSYY-2024–0746). To construct a tumor-bearing mouse model, LLC cells with a good logarithmic growth state were used. Cell suspension was prepared by Suspending 5× 10^6^ cells in PBS buffer (100 μl) and injected subcutaneously into the back of each mouse. To inhibit thyroid uptake of free ^131^I, tumor-bearing animals were fed a 1% KI solution 3 d before the start of all imaging or therapy tests and continued until the end of the experiments.

### In vivo antitumor study

When the tumor grew to 50 mm^3^, the tumor-bearing mice were randomly divided into 4 groups (*n* = 7). The day of grouping was recorded as day 1, and the drug administration began. The control group and treatment group were given 100 μl of PBS buffer, ^131^I (500 μCi), Mn/SAE (300 μg), and ^131^I-Mn/SAE@M (500 μCi), respectively. The method of administration was caudal venous injection. The weight of tumor-bearing mice was measured every other day, and the maximum length (L) and width (W) of tumors were measured with vernier calipers. The tumor volume (V) is calculated as follows: V = (L × W^2^)/2. Fourteen days after treatment, the mice were euthanized, and the tumors were dissected, weighed, and photographed.

In parallel, to assess antitumor immune responses, tumor tissues and spleens were collected at the end of treatment for further immunological analysis. Single-cell suspensions were collected and digested, then stained with the corresponding antibodies. Activated T cells (FITC-anti-mouse CD3 and APC-anti-mouse CD8 antibodies), dendritic cells (DCs: FITC-anti-mouse CD11c, PE-anti-mouse CD86, and APC-anti-mouse CD80 antibodies), regulatory T cells (Tregs: PE-Cy7-anti-mouse CD4 and PE-anti-mouse Foxp3 antibodies), marrow-derived suppression cells (MDSCs: FITC-anti mouse CD11b, PE-Cy7- anti mouse GR1, APC-anti mouse Ly6C, PE-anti mouse Ly6G), tumor-associated macrophages (TAMs: FITC-anti mouse CD11b, APC-anti mouse-F4/80, PE-anti mouse-CD86, PE-Cy7-anti mouse-CD206), and immune effector memory T cells (T_EM_: CD3^+^CD8^+^CD44^+^CD62L^−^) were eventually identified by flow cytometry. A more detailed description of the method is provided in Additional file 1: Materials and methods.

### Statistical analysis

Details regarding the sizes of groups, the number of replicates, and the definitions of mean values and error bars are meticulously detailed in the legends accompanying each figure. Statistical analyses were conducted using the GraphPad Prism 9.0 software suite. To compare the differences between two distinct groups, we employed either a two-tailed unpaired *t*-test or the Mann-Whitney *U* test. For comparisons involving three or more groups, we utilized one-way analysis of variance (ANOVA) followed by Tukey’s multiple comparison test to conduct post-hoc analysis. All reported data are presented as mean ± standard deviation (SD). *P* < 0.05 was considered to indicate statistical significance.

## Results

### Synthesis and characterization of Mn/SAE and ^131^I-Mn/SAE@M

**Fig. 1:**
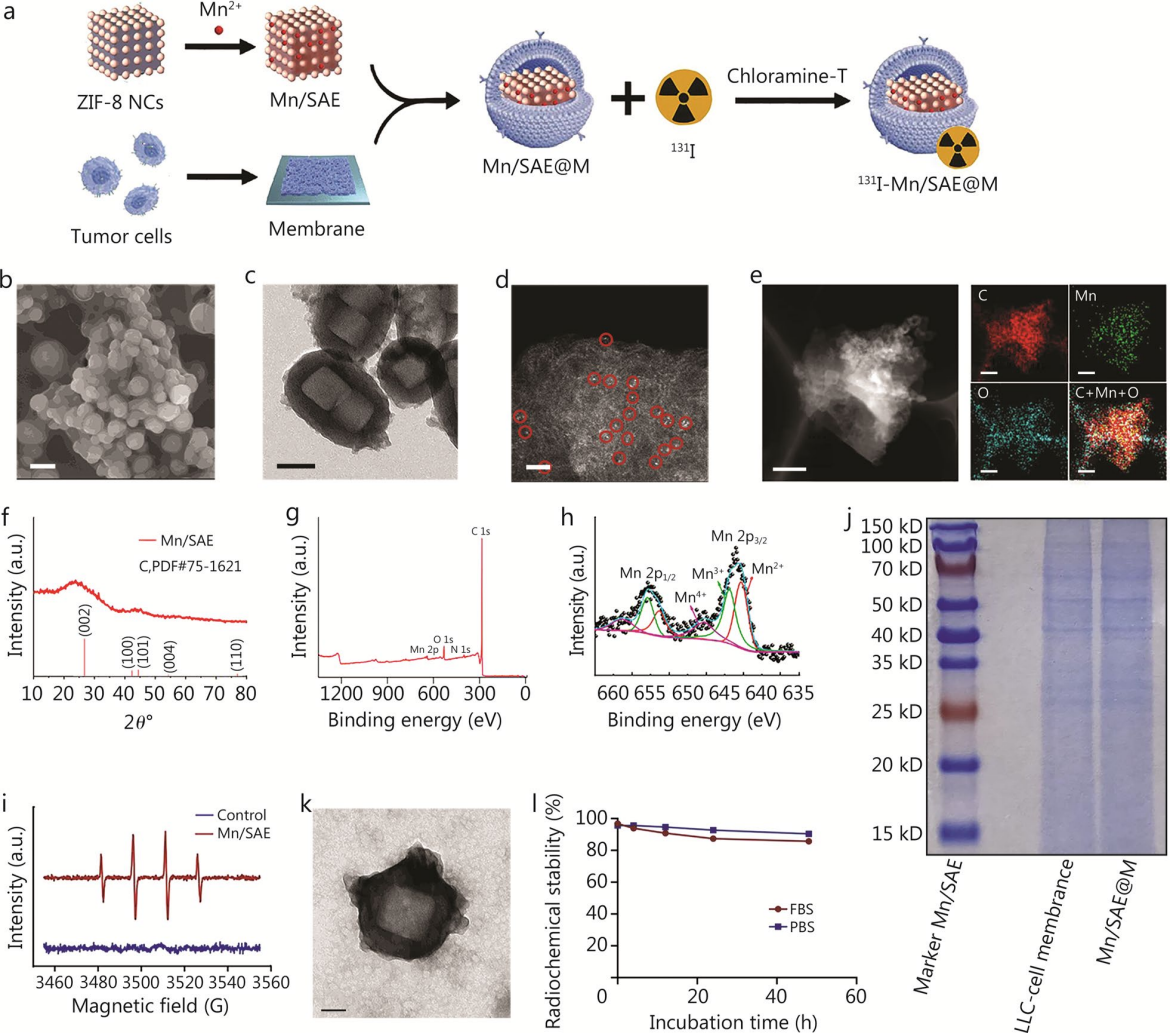
Synthesis and characterization of Mn/SAE and ^131^I-Mn/SAE@M. **a** Schematic illustration of the formation of Mn/SAE@M and ^131^I-Mn/SAE@M. SEM (**b**, scale bar = 200 nm), TEM (**c**, scale bar = 100 nm), and HAADF STEM (**d**, scale bar = 2 nm) images of Mn/SAE, wherein bright dots (indicated by red circles) indicate the dispersive single iron atoms. **e** Atom mapping images of C, Mn, and O of prepared Mn/SAE. Scale bar = 400 nm. **f** XRD patterns of Mn/SAE. **g** XPS survey spectra of Mn/SAE. **h** XPS spectra for Mn 2p in Mn/SAE. **i** ESR spectra of ·OH using DMPO as a capturing agent. **j** SDS-PAGE gel analysis of Mn/SAE nanoparticles, LLC-cell membranes, and Mn/SAE@M nanoparticle protein contents. **k** TEM image of Mn/SAE@M. Scale bar = 100 nm. **l** The radiolabeling stability analysis of ^131^I-Mn/SAE@M incubated in PBS and FBS (37 °C) for 4, 12, 24, and 48 h. Data are expressed as mean ± SD (*n* = 3). SD standard deviation, DMPO dimethylpyridine nitroxide, ESR electron spin resonance, FBS fetal bovine serum, HAADF STEM high-angle annular dark-field scanning transmission electron microscopy, ·OH hydroxyl radicals, Mn/SAE manganese single-atom nanozymes, SEM scanning electron microscopy, SDS-PAGE sodium dodecyl sulfate–polyacrylamide gel electrophoresis, TEM transmission electron microscopy, XPS X-ray photoelectron spectroscopy, XRD X-ray powder diffraction, ZIF-8 NCs zeolitic imidazolate frame-8 nanocubes, LCC Lewis lung carcinoma

The synthesis process of Mn/SAE and ^131^I-Mn/SAE@M was illustrated (Fig. 1a). In brief, the TA solution was utilized to etch ZIF-8 NCs for obtaining hollow structures, followed by ion exchange to impregnate Mn^2+^ and subsequent pyrolysis to yield Mn/SAE. Ultrasound-assisted nanoencapsulation method was used to coat extracted cancer cell membranes onto Mn/SAE, and the Chloramine-T method was adopted to radiolabel ^131^I on Mn/SAE@M, obtaining the ultimate ^131^I-Mn/SAE@M radiopharmaceuticals. The successful synthesis of ZIF-8 NCs was confirmed by EDS mapping (Additional file 1: Fig. S1), which demonstrated a uniform distribution of key elements (C and N), indicating the homogeneity and compositional integrity of the nanocrystals. Mn/SAE exhibited a hollow nanocubic structure with 100 to 200 nm in diameter (Fig. 1b, c). High-resolution and atom mapping images confirmed the presence of Mn-Nx active centers (Fig. 1d, e), akin to other metal-Nx active centers [17, 18]. X-ray powder diffraction (XRD) data showed 2 distinct diffraction peaks at approximately 25.3° and 43.1° corresponding to the (002) and (101) planes of graphitic carbon, demonstrating successful synthesis of Mn/SAE on graphitic carbon support (Fig. 1f). The co-existence of multivalences (+ 2 and + 3) in Mn atoms was observed (Fig. 1g, h), indicating the oxidation state and N coordination with Mn atoms was also decided since the typical peak at approximately 399.7 eV that corresponds to tertiary nitrogen or metal-nitrogen coordination is observed (Additional file 1: Fig. S2). These high oxidation states dictated the high multienzymatic catalysis of Mn-Nx active centers for ROS production, as indicated by electron spin resonance (ESR) spectra of hydroxyl radicals (Fig. 1i).

Objective to NSCLC with poor immunogenicity, the homologous LLC cell membrane was used to camouflage Mn/SAE. Protein gel electrophoresis analyzed the markers of LLC cell membrane, and Mn/SAE alone did not show any proteins yet, while Mn/SAE@M showed approximately identical bands with LLC cell membrane, including positions and clearness (Fig. 1j). The fourier transform infrared spectroscopy (FTIR) and X-ray photoelectron spectroscopy (XPS) results also revealed successful formation of Mn/SAE@M, because the C = O stretching vibration in FTIR spectra and N–O, C = O and C-O in N 1 s and O 1 s XPS spectra are clearly observed while ZIF-8 has no O atoms (Additional file 1: Fig. S3). Additionally, further electron beam irradiation-induced fusion on the surface of Mn/SAE@M also unveiled the successful coating of Mn/SAE with LLC cell membrane (Fig. 1k; Additional file 1: Fig. S4). LLC cell membrane coating armed Mn/SAE with high dispersity, thus resulting in smaller hydrated dynamic size (Additional file 1: Fig. S5a, but the negative cell membrane rendered the surface potential of Mn/SAE more negative (Additional file 1: Fig. S5b). Through the Chloramine-T method, ^131^I was successfully conjugated onto the LLC cell membrane on Mn/SAE@M (Additional file 1: Fig. S6), and ^131^I failed to shed from the obtained ^131^I-Mn/SAE@M, exhibiting a high stability (Additional file 1: Fig. S7). The LLC cell membrane coating and ^131^I chelation have negligible influences on particle size since the particle size of ^131^I-Mn/SAE@M remained approximately 100–200 nm (Additional file 1: Fig. S6), which is appropriate for maintaining enzymatic activity and colloidal stability in solution and ensuring the feasibility of intravenous administration via tail vein when used in vivo. Additionally, such radiolabeled ^131^I-Mn/SAE@M exhibited a radiochemical purity of up to 95% and showed a prolonged radiolabeling stability (80% retention after 48 h) under the conditions of PBS and FBS at 37 °C (Fig. 1l), which is sufficient for killing tumors.

### Multienzyme activity test

**Fig. 2:**
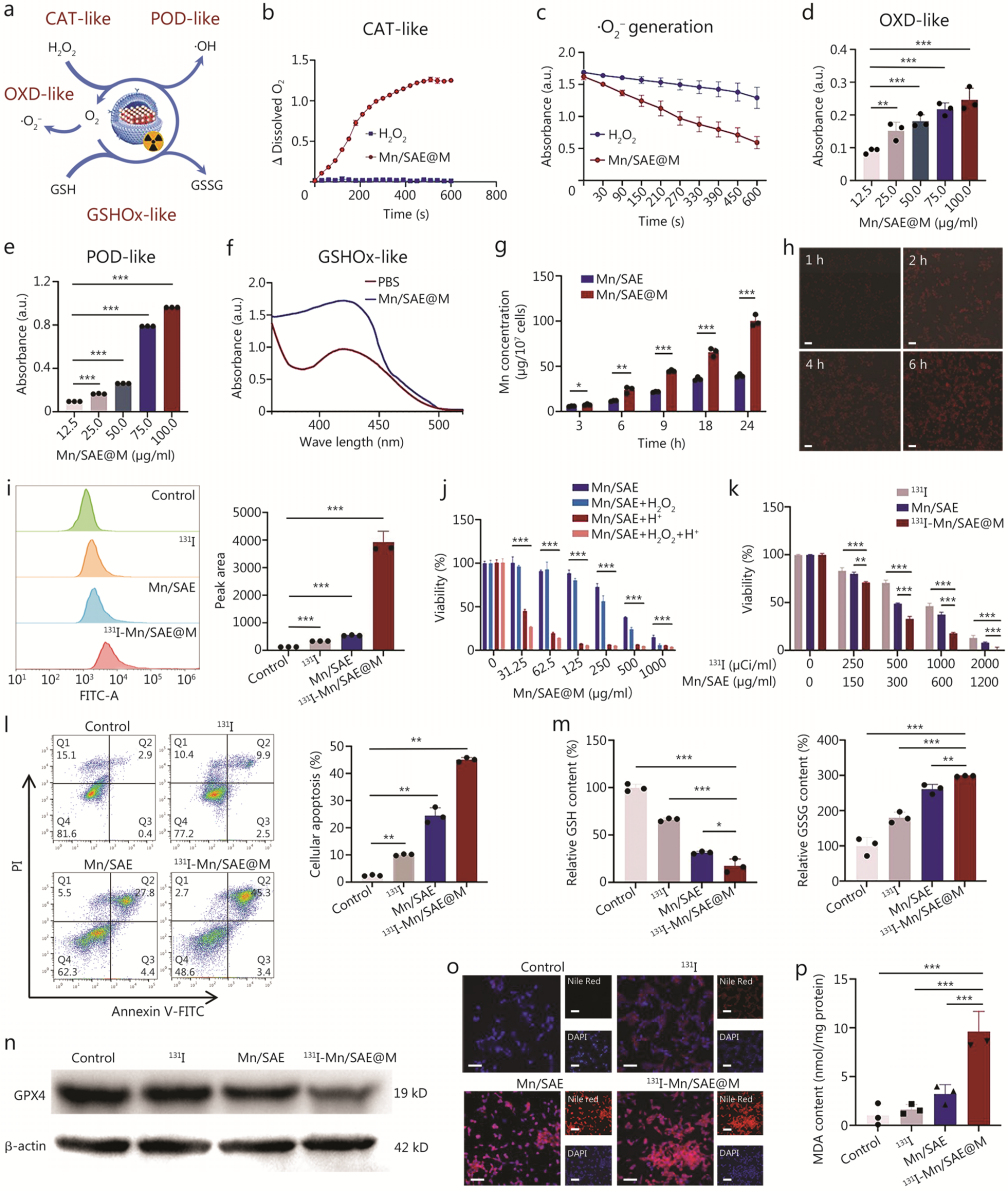
Multienzyme-mimicking activity, antitumor, and lipid metabolism disruption analysis based on ^131^I-Mn/SAE@M in vitro. **a** Schematic of a multienzyme-mimicking biocatalytic process. **b** Dissolved oxygen (O_2_) levels of Mn/SAE@M in H_2_O_2_ solution for monitoring the CAT-mimicking activity. **c** The·O_2_^−^ Generation efficiency is measured by bleaching the DPBF absorbance at 420 nm with H_2_O_2_ (2 mmol/L). **d** TMB assay for measuring OXD-like activity of the Mn/SAE@M at different Mn/SAE@M concentrations (1.0 mmol/L TMB, pH = 5.0, 2.5 mmol/L H_2_O_2_). **e** TMB assay for measuring POD-like activity of the Mn/SAE@M at different concentrations of Mn/SAE@M (1.0 mmol/L TMB, 2.5 mmol/L H_2_O_2_, pH = 5.0). **f** UV–vis spectra of DTNB after incubation with Mn/SAE@M for assessing GSHOx-like activity. **g** Intracellular Mn accumulation at different time points after treatment of LLC cells with Mn/SAE or Mn/SAE@M was measured by ICP-MS. **h** Uptake of ^131^I-Mn/SAE@M by LLC cells at different times by CLSM. Scale bar = 50 μm. **i** Representative flow cytometry images of ROS in LLC cells stained with DCFH-DA after different treatments. **j** Viability of LLC cells treated with Mn/SAE in the absence or presence of H_2_O_2_ (2.5 mmol/L). The assays were performed at pH 7.4 and pH 6.0, respectively. **k** The cytotoxic effect of ^131^I, Mn/SAE, and ^131^I-Mn/SAE@M with gradient activity on LLC cells for 24 h was evaluated by CCK-8 assays (*n* = 3). **l** Flow cytometry patterns and data of LLC cells stained with PI and annexin V-FITC after various treatments for determining the apoptosis level (*n* = 3). **m** GSH and GSSG levels in LLC cells after different treatments. **n** Western blotting analysis of GPX4 protein levels after different treatments. **o** CLSM images for visualizing lipid droplets (red) in LLC cells after different treatments with Nile Red staining. Scale bar = 100 μm. **p** The MDA levels in LLC cells after different treatments. Data are expressed as mean ± SD (*n* = 3). ^*^P < 0.05, ^**^P < 0.01, ^***^P < 0.001. SD standard deviation, CAT catalase, CCK-8 cell counting kit-8, CLSM confocal laser scanning microscopy, DTNB 5,5'-dithiobis-(2-nitrobenzoic acid), GPX4 glutathione peroxidase 4, GSH glutathione, ICP-MS inductively coupled plasma-mass spectrometry, ROS reactive oxygen species, MDA malondialdehyde, MFI mean fluorescence intensity, OXD oxidase, POD peroxidase, PI propidium iodide, TMB 3,3',5,5'-tetramethylbenzidine, LLC Lewis lung carcinoma, GSHOx glutathione oxidase, ^131^I iodine-131, Mn/SAE manganese-based single-atom nanozyme, ^131^I-Mn/SAE@M iodine-131-membrane-coated manganese single-atom nanozymes, DAPI 4'-6-diamidino-2-phenylindole

Mono-distributed Mn-Nx active centers determine the high multienzyme-mimicking capabilities of Mn/SAE@M radiopharmaceuticals including CAT-l, OXD-, POD-, and GSHOx-like activities (Fig. 2a). It was validated that Mn/SAE@M holds high CAT-like activity to effectively catalyze the decomposition of H_2_O_2_ into O_2_, as evidenced by the continuous O_2_ bubbling (Additional file 2: Video S1) and dissolved O_2_ elevation (Fig. 2b). Compared to the control group, Mn/SAE@M treatment led to a visibly faster reaction rate and a notable increase in dissolved O_2_ levels within minutes, indicating efficient catalytic conversion of H_2_O_2_. The rapid O_2_ generation highlights the strong CAT-mimicking capability of Mn/SAE@M under physiological conditions.

During OXD-like catalysis, Mn/SAE@M catalyzed the transfer of electrons to O_2_ and gave birth to superoxide radicals (Fig. 2c; Additional file 1: Fig. S8). Furthermore, 3,3',5,5'-tetramethylbenzidine (TMB) was used as a chromogenic substrate to discern superoxide radicals, and results showed that OXD-catalytic superoxide radicals by Mn/SAE@M can oxidize colorless TMB into blue products with a characteristic absorbance peak at 650 nm (Additional file 1: Fig. S9). Higher Mn/SAE@M concentration favored more superoxide radicals’ birth to color TMB (Fig. 2d; Additional file 1: Fig. S9a), revealing that the high OXD-like catalysis activity of Mn/SAE@M catalyzes plenty of superoxide radicals. Especially under the same Mn/SAE@M concentration, no matter how many TMB were added, they are catalyzed by Mn/SAE@M and converted into blue products (Additional file 1: Fig. S9b). Intriguingly, the OXD-like activity of Mn/SAE@M was significantly enhanced under weakly acidic conditions (Additional file 1: Fig. S9c).

It is known that the POD process can decompose intratumoral H_2_O_2_ to generate hydroxyl radicals by accelerating the Fenton-like reaction, and hydroxyl radicals (·OH) can also color TMB. In the presence of H_2_O_2_, Mn/SAE@M successfully switched colorless TMB to blue products (Additional file 1: Fig. S10). With resembling OXD-like activity, Mn/SAE@M also had high POD-like catalysis activity, and higher concentration favors more ·OH production (Fig. 2e), wherein POD-like activity was boosted under weakly acidic TME (Additional file 1: Fig. S10a). As TMB or H_2_O_2_ concentration increases, more blue products were produced under identical Mn/SAE@M concentration (Additional file 1: Fig. S10b, c). Notably, higher concentrations of Mn/SAE@M received more blue products, meaning the Mn/SAE confers Mn/SAE@M with the robust multienzymatic activities (Additional file 1: Fig. S10d). As well, methylene blue (MB) was used to test the POD-like activity, and identical results with the TMB experiment were obtained, where more hydroxyl radicals were produced via POD-like catalysis of Mn/SAE@M, especially under acidic and H_2_O_2_-rich milieu to degrade MB (Additional file 1: Fig. S11).

Furthermore, 5,5'-dithiobis-(2-nitrobenzoic acid) (DTNB) as an indicator was leveraged to assess GSHOx-like activity of Mn/SAE@M, where Mn/SAE@M had high GSHOx-like activity to oxidize GSH, resulting in the decline at the characteristic absorbance peak value of DTNB (Fig. 2f). The above results unveiled that the high multienzymatic catalysis of Mn/SAE@M successfully produces abundant ROS via disrupting intratumoral redox balance. This is highly preferable for improving ^131^I-mediated TRT since ROS can not only directly kill tumor cells, but also mitigate immunosuppressive TME and enhance immunogenic cell death (ICD).

### Antitumor tests in vitro

Before assessing ^131^I-mediated TRT and Mn/SAE-mediated multienzymatic catalysis for killing LLC, the engulfment test of ^131^I-Mn/SAE@M was implemented. Due to the homologous tropism, the coated LLC cell membrane allowed massive Mn/SAE@M to enter and accumulate in LLC cells, and the prolonged incubation time favored more Mn/SAE@M engulfment by LLC cells with twofold increment (Fig. 2g). Similar results were obtained through direct observation, wherein rhodamine B-labeled ^131^I-Mn/SAE@M was gradually internalized by LLC cells in a time-dependent manner (Fig. 2h).

The high accumulation of ^131^I-Mn/SAE@M radiopharmaceuticals in LLC cells significantly contributed to the most ROS production through ^131^I-mediated TRT and Mn/SAE@M-mediated multienzymatic catalysis (OXD, POD) processes, as evidenced in Fig. 2i and Additional file 1: Fig. S12. To decipher the upregulated types of ROS, DHE, HPF, and MitoPY1 dyes were used to discern and detect ·O_2_⁻, ·OH, and H_2_O_2_, respectively. Consistent with that of total ROS mentioned above, the considerably elevated levels of all 3 species were observed in ^131^I-Mn/SAE@M-treated cells (Additional file 1: Fig. S13), further corroborating the multienzyme-like activity of ^131^I-Mn/SAE@M. This phenomenon could be attributed to Mn/SAE-mediated O_2_ release to provide source materials for ROS production during ^131^I-mediated TRT (Additional file 1: Fig. S14). Excessive intracellular ROS disrupts the redox metabolism, which induces cell damage and even deaths. Since POD- and OXD-like catalytic activities of Mn/SAE for ROS production can be potentiated by H_2_O_2_ and acidic microenvironment, their antitumor outcomes against LLC cells were indeed significantly encouraged by additionally introducing H_2_O_2_ and H^+^ as a function of Mn/SAE concentration (Fig. 2j). This cell counting kit-8 (CCK-8) result promised the excellent tumor progression inhibition in vivo using such Mn/SAE since solid tumor is characterized with acidic and H_2_O_2_-abundant microenvironment. Depending on the most ROS production, ^131^I-Mn/SAE@M exerted the most powerful killing ability to reject the proliferation of LLC tumor cells (Fig. 2k), and the killing outcome displayed a dose-dependent manner.

Flow cytometry were used to differentiate stages of LLC cells after treatments with different samples and co-staining with Annexin V-FITC and propidium iodide (PI). Identical results are obtained that ^131^I alone and Mn/SAE alone triggered cell deaths to some extent, respectively, while their combination in ^131^I-Mn/SAE@M induced the largest percentage of apoptotic cells (above 40%), and all dead cells occupied the late apoptosis zone (Fig. 2l). Furthermore, co-staining analysis with PI and Calcein-AM confirmed the strongest cytotoxicity of ^131^I-Mn/SAE@M on LLC cells (Additional file 1: Fig. S15).

### Lipid metabolism disruption by ^131^I-Mn/SAE@M

Ferroptosis is a form of non-apoptotic cell death characterized by glutathione (GSH) depletion, reduced GPX4 expression, diminished antioxidant capacity, and increased lipid peroxidation [19–21]. In this context, we investigated the tumor ferroptosis and lipid metabolic homeostasis in tumors following treatment with ^131^I-Mn/SAE@M.

Firstly, we observed that ^131^I-Mn/SAE@M effectively depletes enriched GSH and increases GSSG content in tumor cells, during which ROS consumption by GSH was hampered and ROS accumulation was further enlarged (Fig. 2m). Subsequently, the expression of GPX4 in tumor cells post ^131^I-Mn/SAE@M treatment was verified at the cellular level, aligning with the in vivo findings, demonstrating a significant reduction in GPX4 expression (Fig. 2n; Additional file 1: Fig. S16). Adenosine triphosphate (ATP) as the energy source sustains the abnormal proliferation and growth of living cells, which also serves as the fundamental unit to decide subsequent various metabolism processes, such as amino acid, lipid, or glucose metabolism. After different treatments, it was found that ^131^I-Mn/SAE@M posed a significant decline in intracellular ATP levels (Additional file 1: Fig. S17). The JC-1 staining assay also validated that ^131^I-Mn/SAE@M successfully brings about mitochondrial damage to disrupt LLC cell metabolisms and dampen ATP synthesis, which explained why ^131^I-Mn/SAE@M induced the most LLC cell apoptosis or growth arrest (Additional file 1: Fig. S18).

Among various species’ metabolism, lipid metabolism is highlighted since it can directly and indirectly affect immune activities [22, 23]. Our results showed that ^131^I-Mn/SAE@M caused lipid metabolism disruption, and tremendously decreased the content of intratumoral lipid compounds, as evidenced by the Nile Red staining (Fig. 2o). The considerably decreased intratumoral lipid compounds were attributed to the decomposition and oxidation of lipid compounds into malondialdehyde (MDA, a lipid peroxidation product) by abundant ROS produced in ^131^I-Mn/SAE@M (Fig. 2p). These results revealed that ^131^I-Mn/SAE@M indeed produces adequate ROS via ^131^I-mediated TRT and Mn/SAE multienzymatic catalysis to disrupt lipid metabolism process of LLC cells, and caused late LLC cell apoptosis.

### In vivo antitumor survey

**Fig. 3:**
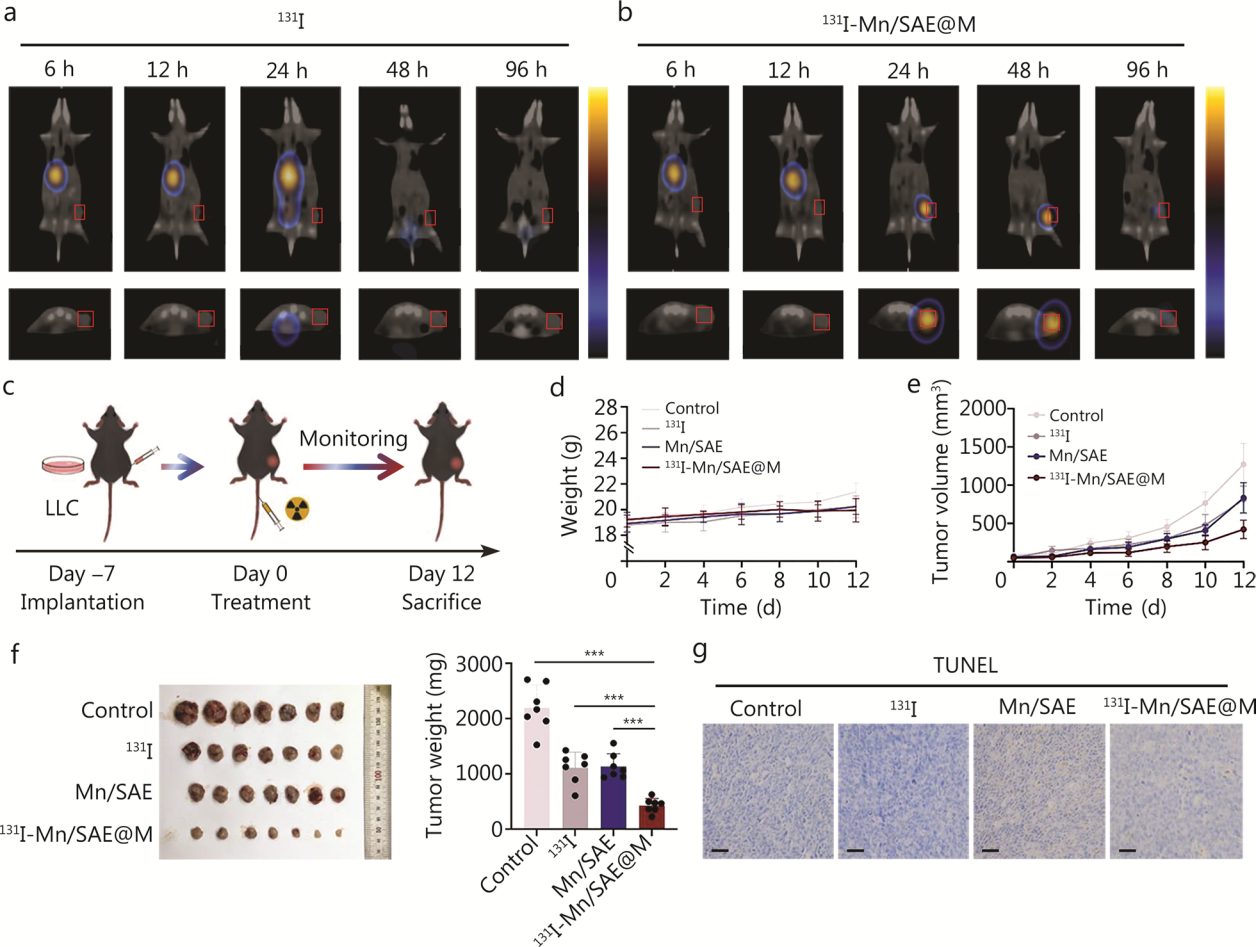
Antitumor survey of ^131^I-Mn/SAE@M in vivo*.* **a** Representative SPECT/CT imaging of LLC tumor-bearing mice post-intravenous injection of ^131^I at different time points. Red boxes highlight the tumor sites in both coronal and transverse sections. **b** Representative SPECT/CT imaging of LLC tumor-bearing mice post-intravenous injection of ^131^I-Mn/SAE@M at different time points. Red boxes highlight the tumor sites in both coronal and transverse sections. **c** Schematic of in vivo antitumor procedures. **d** Body weight variation curves of LLC tumor-bearing mice in corresponding treatment groups (*n* = 7). **e** Average tumor growth curves of LLC tumor-bearing mice in corresponding treatment groups (*n* = 7). **f** Photos of excised tumors and corresponding tumor weights from mice on day 12 after different treatments. **g** Representative optical microscopic images of TUNEL-stained tumor sections in corresponding treatment groups. Scale bar = 50 μm. Data are expressed as mean ± SD (*n* = 7). ^***^P < 0.001. SD standard deviation, LLC Lewis lung carcinoma, ^131^I iodine-131, Mn/SAE manganese-based single-atom nanozyme, ^131^I-Mn/SAE@M iodine-131-membrane-coated manganese single-atom nanozymes, SPECT/CT single-photon emission computed tomography/computed tomography, TUNEL terminal deoxynucleotidyl transferase dUTP nick end labeling

Based on the above inspiring in vitro experiments, ^131^I-Mn/SAE@M for in vivo antitumor therapy can be expected. Especially, it is worth noting that tumor-bearing animals were fed with a 1% KI solution 3 d before starting all in vivo experiments to inhibit thyroid uptake of free ^131^I. Single-photon emission computed tomography/computed tomography (SPECT/CT) imaging was first implemented to evaluate the tumor-targeting capability of ^131^I-Mn/SAE@M and determine the optimal accumulation time at the tumor site. Free ^131^I was found to fail to enter and accumulate in LLC tumor with a low tumor retention rate (Fig. 3a). After labeling on LLC cell membrane coated on Mn/SAE, ^131^I was gradually delivered and primarily concentrated at the site of tumor site after 24 h pose-intravenous injection of ^131^I-Mn/SAE@M, and was maintained up to 96 h (Fig. 3b), validating the powerful tropism ability of homologous LLC cell membrane in ^131^I-Mn/SAE@M to target LLC tumors. Apart from ^131^I, Mn content was also adopted to assess the biodistribution of ^131^I-Mn/SAE@M, and after 24 h post-intravenous injection, there was a significant increase in Mn accumulation within the tumor, and this high-retention stage was Sustained to 48 h (Additional file 1: Fig. S19), which is consistent with the SPECT/CT imaging of ^131^I.

Subsequently, we explored the antitumor activity of ^131^I-Mn/SAE@M in vivo (Fig. 3c), during which mice keep healthy without significant weight loss (Fig. 3d). At the given doses, free ^131^I alone and Mn/SAE alone delay tumor growth, but their inhibition rates are inferior to ^131^I-Mn/SAE@M owning to their insufficient intratumoral accumulation and low ROS production in tumor. The combination of ^131^I with Mn/SAE@M received the largest inhibition rate at any monitoring time (Fig. 3e; Additional file 1: Fig. S20). After resection, the tumors were harvested, and we observed that the size and weight of tumors in mice treated with ^131^I-Mn/SAE@M were much lower than those from other groups (Fig. 3f). H&E results showed that tumor cells in the monotherapy groups (either Mn/SAE or ^131^I alone) retained relatively intact structures, while in the ^131^I-Mn/SAE@M group, severe necrosis was observed and characterized with clear nuclear condensation and cell shrinkage (Additional file 1: Fig. S21). The terminal deoxynucleotidyl transferase dUTP nick end labeling (TUNEL) assay further confirmed these results, where the combination therapy, ^131^I-Mn/SAE@M, led to the most cell apoptosis (Fig. 3g). More significantly, such Mn/SAE-based radiopharmaceuticals had no visible effect on normal tissues and biochemical markers (Additional file 1: Figs. S22, S23), suggested high biocompatibility of ^131^I-Mn/SAE@M for clinical translation.

### In vivo antitumor mechanism analysis

**Fig. 4:**
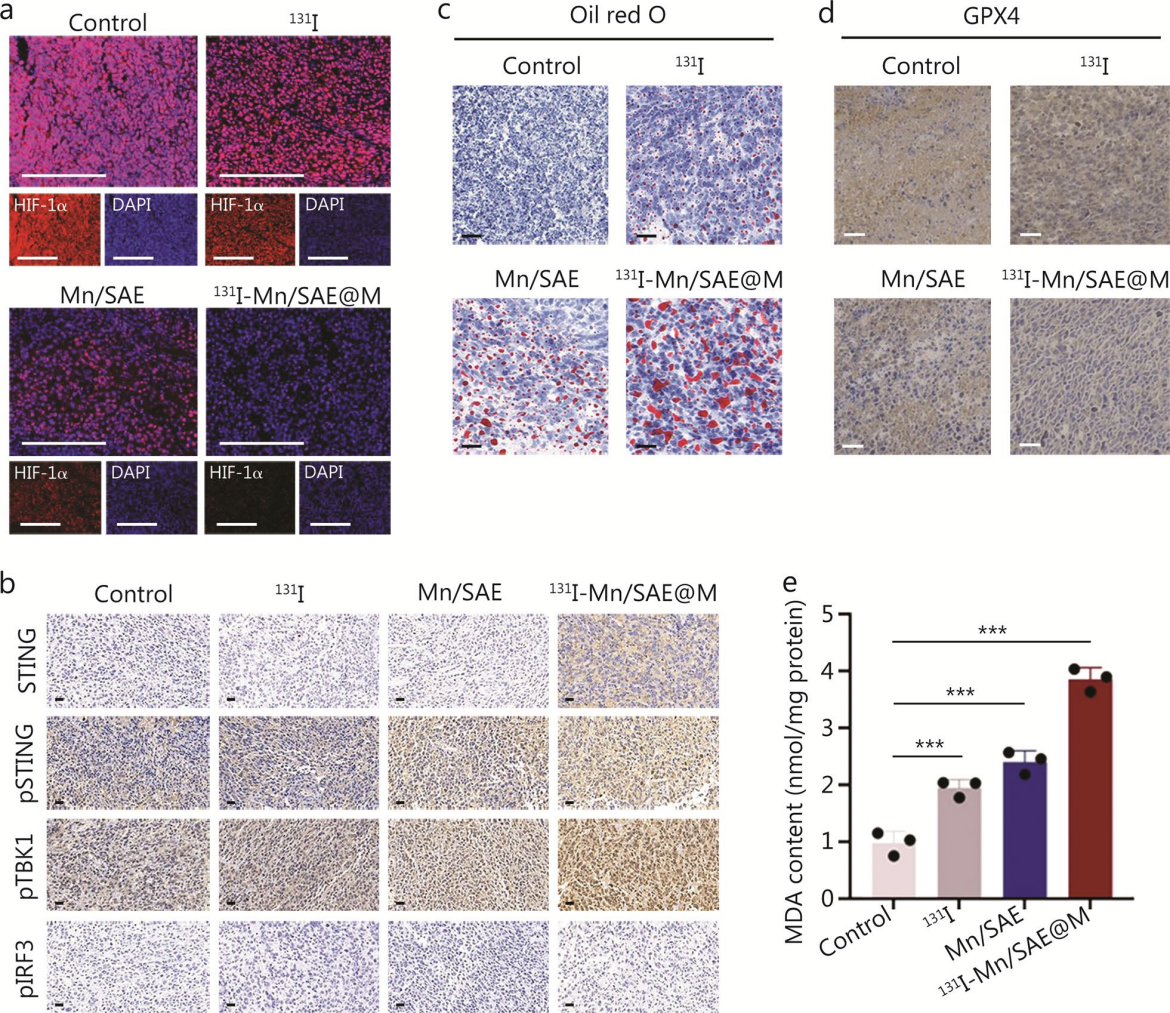
Hypoxia mitigation, STING pathway and ferroptosis activations, and lipid metabolism disruption induced by ^131^I-Mn/SAE@M in vivo. **a** Visualization of hypoxia in tumor sections after different treatments with HIF-1α staining. Scale bar = 100 μm. **b** Representative optical microscopic images of STING, pSTING, pTBK1, and pIRF3-stained tumor sections in corresponding treatment groups. Scale bar = 20 μm. **c** Representative optical microscopic images of lipid droplets (red) in tumor sections after different treatments with Oil red O staining. Scale bar = 50 μm. **d** Representative optical microscopic images of GPX4-stained tumor sections in corresponding treatment groups. Scale bar = 20 μm. **e** The MDA contents in LLC sections after different treatments. Data are expressed as mean ± SD (*n* = 3). ^***^P < 0.001. SD standard deviation, GPX4 glutathione peroxidase 4, MDA malondialdehyde, ^131^I iodine-131, Mn/SAE manganese-based single-atom nanozyme, ^131^I-Mn/SAE@M iodine-131-membrane-coated manganese single-atom nanozymes, HIF-1α Hypoxia-inducible factor-1alpha, DAPI 4'-6-diamidino-2-phenylindole, pSTING phosphorylated cyclic GMP-AMP receptor stimulator of interferon genes, pTBK1 phosphorylated TANK-binding kinase 1, pIRF3 phosphorylated interferon regulatory factor 3

Hypoxia may reshape immunosuppressive TME, rendering the tumor more resistant to ROS therapy [4, 24, 25]. Fortunately, the CAT-like enzymatic catalysis of Mn/SAE produces abundant O_2_ to tremendously mitigate the hypoxic microenvironment. Combining with it, LLC cell membrane tropism-mediated accumulation also encouraged ^131^I-Mn/SAE@M to receive the lowest hypoxia-inducible factor-1alpha (HIF-1α) expression (Fig. 4a). To further understand the action principle, the cyclic GMP-AMP synthase (cGAS)-stimulator of interferon genes (STING) pathway associated with ionizing radiation was monitored. Results proved that STING, phosphorylated STING (pSTING), phosphorylated TANK-binding kinase 1 (pTBK1), and phosphorylated interferon regulatory factor 3 (pIRF3) are up-regulated in tumor tissues after ^131^I-Mn/SAE@M treatments, confirming the activation of the STING signaling pathway (Fig. 4b).

A close association between the activation of the STING pathway and lipid peroxidation has been identified [26]. Therefore, we further explored lipid metabolism after ^131^I-Mn/SAE@M treatments, wherein Oil red O, a fat-soluble dye, was used to bind with and stain neutral triglycerides (TG), lipids, and lipoproteins [27]. A significant increase in red color was observed in the ^131^I-Mn/SAE@M-treated tumor tissues, suggesting a significant increase in the content of lipid droplets (Fig. 4c). Interestingly, immunohistochemical staining for GPX4 in tumor tissues revealed a significant decrease in GPX4 expression after treatment with ^131^I-Mn/SAE@M (Fig. 4d). GPX4 is a key enzyme in preventing lipid peroxidation and inhibiting ferroptosis [19]. Therefore, the decrease in GPX4 suggested that the cellular defense mechanism against ferroptosis was suppressed, potentially enhancing the sensitivity of tumor cells to TRT. Additionally, the expression of MDA, that is the final catabolite of lipid peroxidation triggered by free radicals, reached the highest level (Fig. 4e), indirectly reflecting high lipid peroxidation caused by ^131^I-Mn/SAE@M.

### Lipidomics analysis via RNA sequencing

**Fig. 5:**
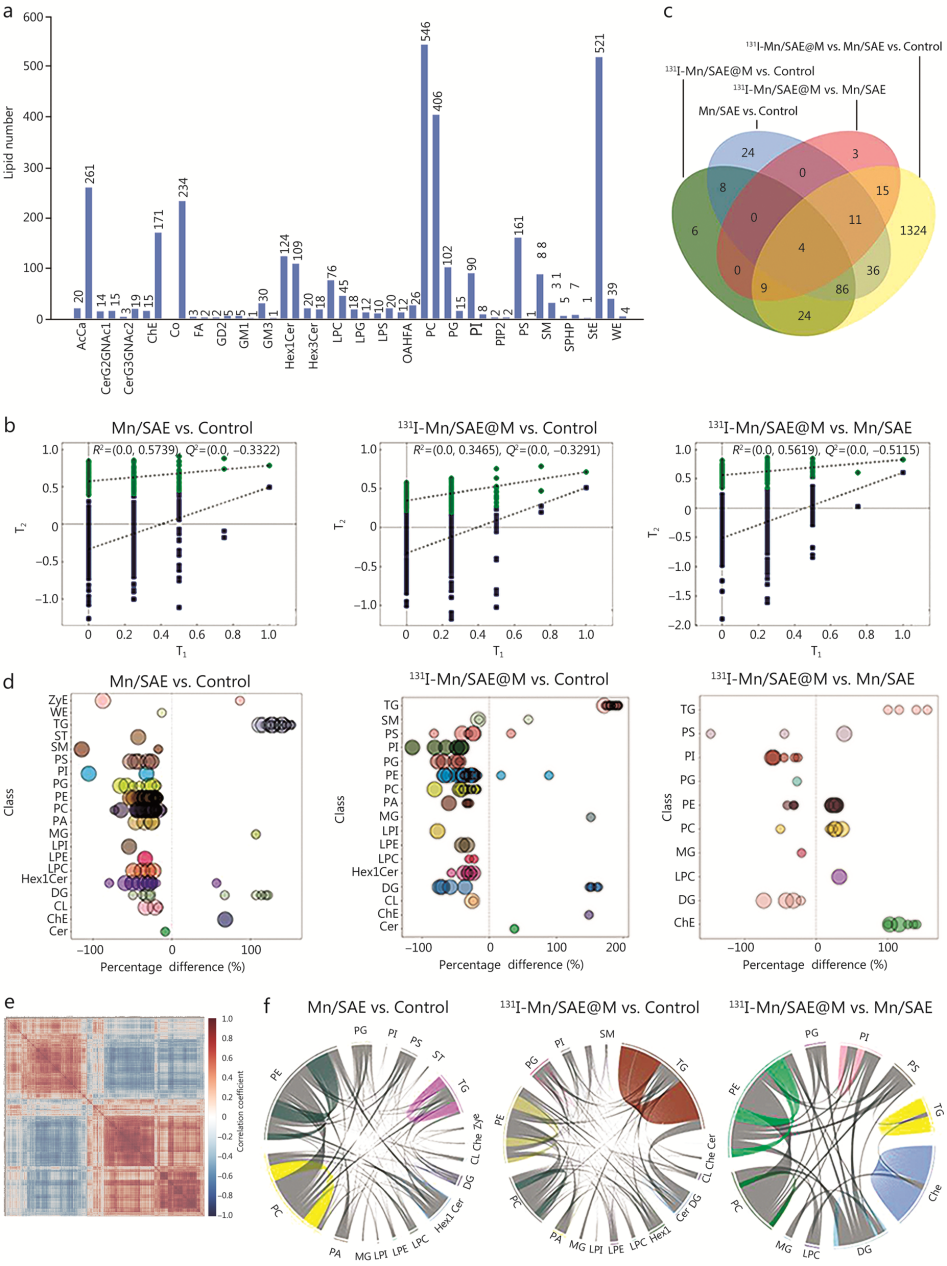
Lipid metabolism analysis of ^131^I-Mn/SAE@M via RNA sequencing. **a** Statistical result for the number of lipid subclasses and the quantity of identified lipid molecules. The two adjacent columns for each lipid class represent the results obtained under positive ion mode and negative ion mode, respectively. **b** Permutation test plot for the OPLS-DA model. The X-axis represents the permutation retention rate, while the Y-axis shows the values of *R*^2^ and *Q*^2^. Green dots represent *R*^2^, and blue dots represent *Q*^2^. **c** Overlap of significantly different lipid molecules identified in various treatment groups. **d** Bubble plots visually display the significant differential lipid molecules screened by different treatment groups. **e** Analysis of the correlation between lipids with significant differences. The color scale represents the correlation coefficient (from –1 to 1), with blue indicating negative correlation and red indicating positive correlation. **f** The correlation between different classes of lipids. PI phosphatidylinositol, PS phosphatidylserine, PE phosphatidylethanolamine, PG phosphatidylglycerol, PC phosphatidylcholine, TG triglyceride, OPLS-DA orthogonal partial least squares discriminant analysis, AcCa acylcarnitine, CerG2GNAc1 monosialylated digalactosylceramide, CerG3GNAc2 trigalactosylceramide, ChE cholesteryl ester, Co coenzyme, FA fatty acid, GD2 disialoganglioside GD2, GM1 monosialoganglioside GM1, GM3 monosialoganglioside GM3, Hex1Cer monohexosylceramide, Hex3Cer trihexosylceramide, LPC lysophosphatidylcholine, LPG lysophosphatidylglycerol, LPS lysophosphatidylserine, OAHFA O-acyl-1-hydroxy fatty acid, PIP2 phosphatidylinositol bisphosphate, SM sphingomyelin, SPHP sphingosine-1-phosphate, StE sterol ester, WE wax ester

Beyond the above mechanism investigation at the protein level, lipidomics analysis was employed to assess the lipid metabolic homeostasis in tumors treated with ^131^I-Mn/SAE@M. Herein, we identified significantly different expressions of 48 lipid classes and 3308 lipid molecules across the categories through positive and negative ion mode identification. In Fig. 5a, the X-axis represents the lipid classes, while the Y-axis indicates the number of identified lipid molecules within each class. Unlike polar metabolites such as amino acids and nucleotides, lipid function studies are primarily conducted at the class level, and different lipid classes exhibit distinct biological functions [28]. By comparing the expression differences in lipid classes among different treatment groups, important lipid classes potentially involved in related biological processes can be further identified (Additional file 1: Fig. S24). Subsequently, principal component analysis (PCA) was performed on lipid molecules. The model showed good reliability and stability based on sevenfold cross-validation, with R^2^X (the proportion of variance in the X matrix explained by the model) values of 0.894 (control group vs. ^131^I-Mn/SAE@M group), 0.868 (Mn/SAE group vs. ^131^I-Mn/SAE@M group), 0.888 (control group vs. Mn/SAE group), and 0.874 (control group vs. Mn/SAE group vs. ^131^I-Mn/SAE@M group), indicating a robust model for distinguishing group differences (Additional file 1: Fig. S25). To avoid overfitting in the supervised model during model construction, a permutation test was first applied to validate the model’s effectiveness (Fig. 5b), and the decrease in the permutation retention rate and the gradual decline of *R*^2^ and *Q*^2^ in the random model confirm no overfitting and high robustness of the original model. The variable importance for the projection (VIP) values obtained from the orthogonal partial least squares discriminant analysis (OPLS-DA) model are available for identifying biologically meaningful differential lipid molecules. The experiment employed OPLS-DA VIP > 1, *P* < 0.05 as the criterion for significant differential lipid molecules selection. Figure 5c displayed the overlap of significant differential lipid molecules identified across comparison groups, and Fig. 5d showed the significant differential lipid molecules between each pair of comparison groups. These results unveiled that ^131^I-Mn/SAE@M indeed interferes with lipid metabolism.

To evaluate the rationality of the differential lipids, we used significantly differentially expressed lipids to perform hierarchical clustering. Results indicated that, compared to the control group, the ^131^I-Mn/SAE@M group and Mn/SAE group exhibit a notable reduction in the content of lipid types such as phosphatidylinositol (PI), phosphatidylserine (PS), phosphatidylethanolamine (PE), phosphatidylglycerol (PG), phosphatidylcholine (PC), and a significant increase in TG subclass lipids (Additional file 1: Fig. S26). Correlation analysis could help measure the metabolic proximity between significant differential lipids (VIP > 1, *P* < 0.05), further shedding light on the relationships among lipids during biological state changes. The correlation analysis results were uncovered using a correlation clustering heatmap, which manifests functional relevance and synthetic conversion between different lipids. In Fig. 5e, red indicates positive correlation and blue indicates negative correlation between significantly altered lipid species (VIP > 1,* P* < 0.05). Lipid molecules with strong correlations tended to cluster together, suggesting potential functional relevance or shared biosynthetic pathways.

To more intuitively uncover the co-regulation relationships among lipids, the lipid-lipid correlation matrix was transformed into a chord diagram. Here, we presented the correlation of lipid molecule pairs with a correlation coefficient *|r|*> 0.8 and *P* < 0.05. TG, PE, PC, and other lipid subclasses in ^131^I-Mn/SAE@M group exhibited more pronounced correlations compared to those in the control group (Fig. 5f). Besides lipid content, the chain length and saturation of lipids are also crucial factors affecting lipid function since they can influence the thickness and fluidity of cell membrane, and regulate the activity and function of related lipid transport proteins and target proteins [29, 30]. After aggregating lipid molecules, we analyzed the content differences in lipids with different carbon chain lengths within each class. It was found that PC saturation matters cell division, migration, and signal transduction in the course of disease occurrence and stress response through influencing cell membrane fluidity (Additional file 1: Figs. S27, S28). The above data shed light on that such camouflaged membrane-bridged Mn/SAE-based radiopharmaceuticals disrupt lipid metabolism, which is highly desirable for evoking antitumor immunity.

### Immune response activation by ^131^I-Mn/SAE@M

**Fig. 6:**
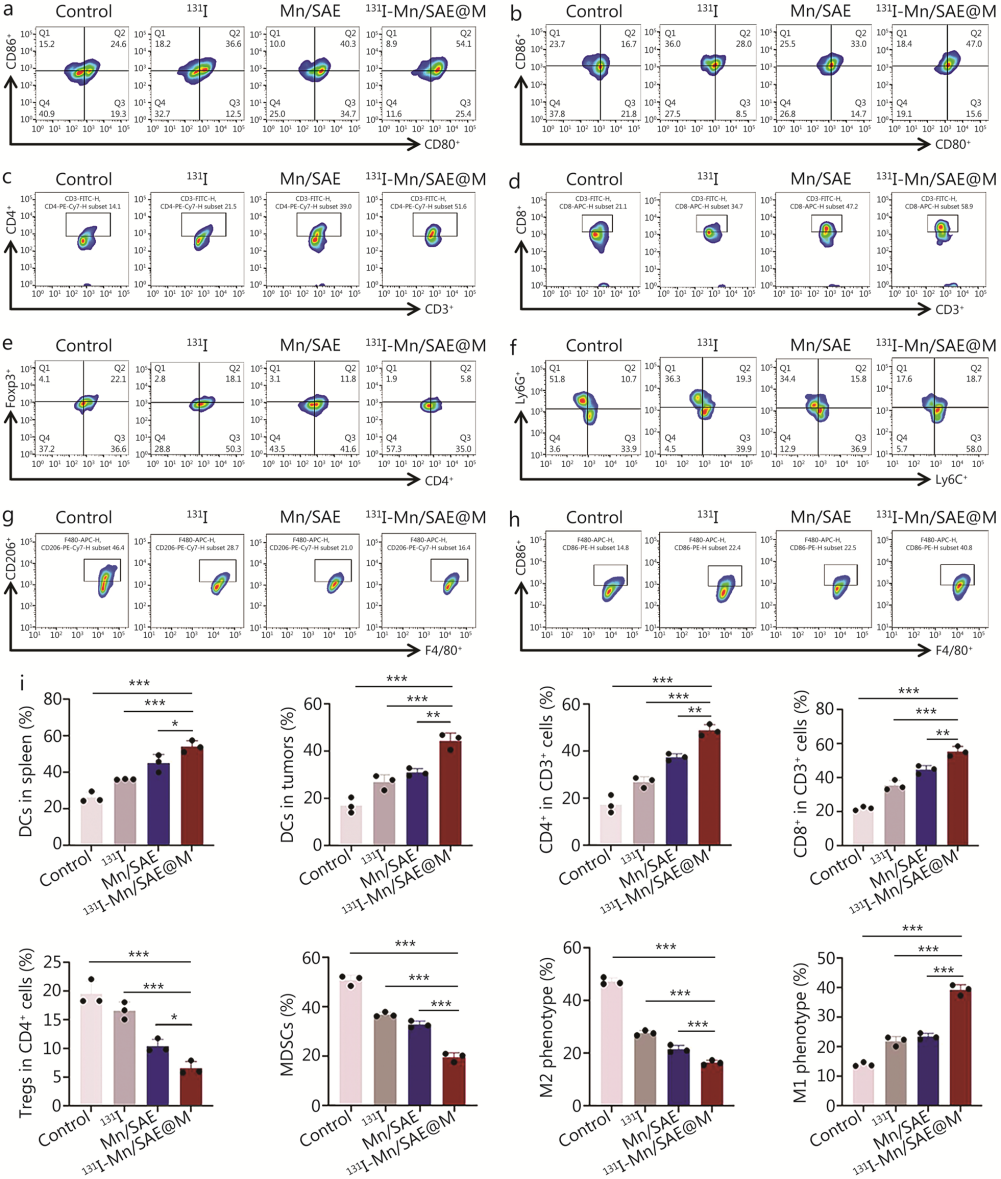
Immune response activation after treatment with ^131^I-Mn/SAE@M. **a** Representative flow cytometry plots showing DCs maturation in spleen from different groups. **b** Representative flow cytometry plots showing DCs maturation in tumors from different groups. **c** Representative flow cytometry plots showing CD4^+^ T cells in tumors from different groups. **d** Representative flow cytometry plots showing CD8^+^ T cells in tumors from different groups. **e** Representative flow cytometry plots showing Tregs in tumors from different groups. **f** Representative flow cytometry plots showing MDSCs in tumors from different groups. **g** Representative flow cytometry plots showing M2 phenotype TAMs in tumors from different groups. Representative flow cytometry plots showing M1 phenotype TAMs in tumors from different groups (**h**) and the statistical data (**i**) (*n* = 3). Data are expressed as mean ± SD (*n* = 3). ^*^P < 0.05, ^**^P < 0.01, ^***^P < 0.001. SD standard deviation, DCs dendritic cells, MDSCs marrow-derived suppression cells, Tregs regulatory T cells, TAMs tumor-associated macrophages, ^131^I iodine-131, Mn/SAE manganese-based single-atom nanozyme, ^131^I-Mn/SAE@M iodine-131-membrane-coated manganese single-atom nanozymes

The damage-associated molecular patterns (DAMPs) released from cancer cells play a crucial role in activating immune responses, and here we monitored the related indicators, including calreticulin (CRT), high mobility group box 1 (HMGB1), and ATP. A significant increase in CRT and HMGB1 of tumor tissues after 7 d of ^131^I-Mn/SAE@M treatment was found, indicating the initiated ICD pathway (Additional file 1: Fig. S29a). Additionally, serum ATP contents measured by ELISA were significantly elevated in the ^131^I-Mn/SAE@M group (Additional file 1: Fig. S29b). These results demonstrated that Mn/SAE@M effectively initiates ICD, which will benefit immune responses. To verify it, we dissected tumor tissues and spleens for flow analysis after 7 d. We discovered that ^131^I-Mn/SAE@M promotes the maturation of DCs within the spleen (Fig. 6a, i; Additional file 1: Fig. S30a), resembling DCs in tumors that highly express DC maturation marker, i.e., CD86 (Fig. 6b, i; Additional file 1: Fig. S30b). Given that the maturation of DCs can mediate downstream immune responses by regulating the activation and expansion of T cells, we further assessed helper T cells (CD4^+^ T cells) and cytotoxic T lymphocytes (CD8^+^ T cells) in the tumors. Compared to control, significant increases in CD4^+^ T and CD8^+^ T cell infiltrations were observed in ^131^I-Mn/SAE@M group (Fig. 6c, d, i; Additional file 1: Fig. S30c, d).

Beyond immune response activation, the attenuation of immunosuppressive TME, including the poor infiltration and depletion of CTLs and DCs, and the recruitment of immunosuppressive cells such as MDSCs, TAMs, and Tregs, can also be anticipated after ^131^I-Mn/SAE@M treatment. It was clearly found that the infiltration of Tregs in tumor was considerably suppressed (Fig. 6e, i; Additional file 1: Fig. S30c), and MDSCs (Fig. 6f, i; Additional file 1: Fig. S30e) and M2 phenotype macrophages (Fig. 6g, i; Additional file 1: Fig. S30f) were dramatically reduced, while M1 phenotype TAMs were significantly increased in ^131^I-Mn/SAE@M (Fig. 6h, i; Additional file 1: Fig. S30g), suggesting M2 phenotype TAMs repolarization into M1 ones is encouraged. Coincidentally, the secretions of some cytokines, including interleukin-10 (IL-10) and transforming growth factor-β (TGF-β) in the serum, were reduced after^131^I-Mn/SAE@M treatment, while IL-12 secretion was promoted (Additional file 1: Fig. S31). These results witness that ^131^I-Mn/SAE@M radiopharmaceuticals exert robust effects on immunity regulation, where they not only activate and potentiate the antitumor immune responses, but also dampen the immunosuppression.

To investigate the temporal correlation between lipid metabolism disruption and antitumor immunity, we conducted a series of in vivo experiments (Additional file 1: Fig. S32). Specifically, we analyzed the infiltration levels of immune cells, including DCs, CD4⁺ T cells, CD8⁺ T cells, MDSCs, M2 phenotype TAMs, and M1 phenotype TAMs (Additional file 1: Figs. S32a-g, S33) of tumors under 1, 3, and 7 d post-treatment. Simultaneously, we measured the lipid droplet content in the tumor tissues by Oil red O staining (Additional file 1: Fig. S32h). On the first day post-treatment, both immune indicators and lipid metabolism markers exhibited no significant changes, probably due to the insufficient accumulation at the tumor site. However, on days 3 and 7, consistent changes in immune cell infiltration and lipid droplet content suggested that the induction of antitumor immunity through lipid metabolism disruption is likely a dynamic and interconnected process rather than a strictly staged event.

**Fig. 7:**
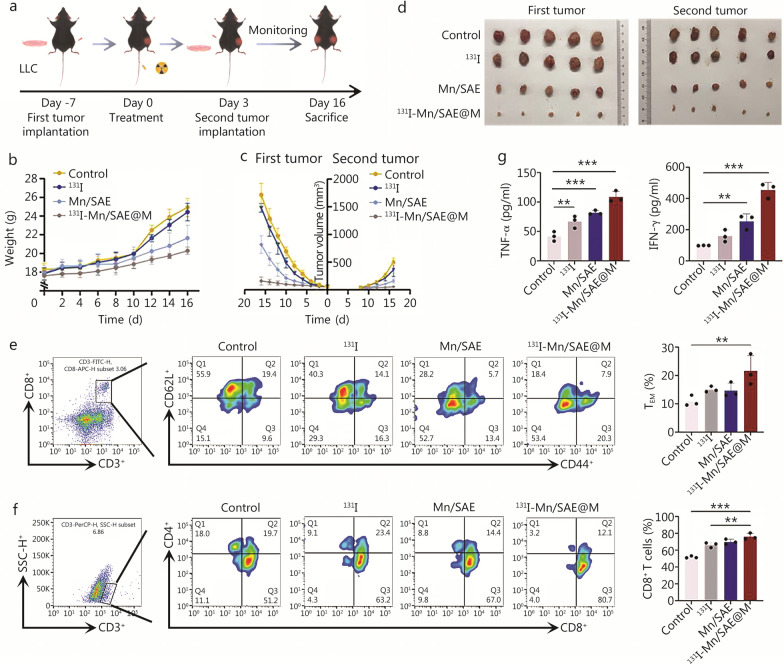
Second tumor inhibition by ^131^I-Mn/SAE@M. **a** Schematic of in vivo antitumor procedures in this bilateral tumor-bearing model. **b** Body weight variation curves of LLC tumor-bearing mice in the corresponding treatment groups (*n* = 5). **c** Average tumor growth curves of LLC tumor-bearing mice in the corresponding treatment groups (*n* = 5). **d** Photos of representative excised first and second tumors from the mice after 16 d in the corresponding treatment groups (*n* = 5). **e** Flow cytometric analysis of immune effector memory T cells (T_EM_) from the spleens of mice after different treatments (*n* = 3). **f** Flow cytometric analysis of T cells from second tumors of mice after different treatments (*n* = 3). **g** Expression levels of serum cytokines, including TNF-α and IFN-γ, in the mice after different treatments (*n* = 3). Data are expressed as mean ± SD. ^**^P < 0.01, ^***^P < 0.001. SD standard deviation, TNF-α tumor necrosis factor-α, IFN-γ interferon-γ, LLC Lewis lung carcinoma, ^131^I iodine-131, Mn/SAE manganese-based single-atom nanozyme, ^131^I-Mn/SAE@M iodine-131-membrane-coated manganese single-atom nanozymes

To further investigate the effect of ^131^I-Mn/SAE@M in Generating immune memory to inhibit distant tumors, a bilateral tumor-inoculated model was established, where the second tumor was inoculated after 3 d post-treatment of first tumor (Fig. 7a). During treatment, mice remained safety (Fig. 7b). The growth curves of the first and second tumors showed that the tumors in the control and ^131^I groups grow rapidly, and tumors in the Mn/SAE group grow relatively slowly (Fig. 7c; Additional file 1: Fig. S34). By contrast, the slowest growth rate occurred in the ^131^I-Mn/SAE@M group (Fig. 7c; Additional file 1: Fig. S34), demonstrating the excellent therapeutic effect of ^131^I-Mn/SAE@M. After 16 d post-treatment, the intact tumor tissues were dissected. The weights of both first tumors and second tumors in the ^131^I-Mn/SAE@M treatment group were also the lowest (Fig. 7d). Simultaneously, T_EM_ (CD3^+^CD8^+^CD44^+^CD62L^−^) in the spleen were tacked. The activation level of T_EM_ cells significantly increased in the ^131^I-Mn/SAE@M group, confirming the presence of immune memory in the treated mice (Fig. 7e; Additional file 1: Fig. S35a). The activated memory effect aroused CD8^+^ T cells to repress the second tumor, which resulted in the increased infiltration of CD8^+^ T cells in the second tumor (Fig. 7f; Additional file 1: Fig. S35b). In addition, increased secretions of tumor necrosis factor-alpha (TNF-α) and interferon-gamma (IFN-γ) into blood were found in the ^131^I-Mn/SAE@M-treated group (Fig. 7g). All these results indicated that ^131^I-Mn/SAE@M treatment realized CAT-like catalysis-unlocked O_2_ release for mitigating hypoxic TME, OXD-/POD-like catalysis and TRT for expediting ROS production, GSHOx-like catalysis for enabling GSH depletion and ROS enrichment. All these action rationales induced ferroptosis and reprogrammed the hemostasis of lipid metabolism and redox balance to activate the cGAS-STING pathway and potentiate the antitumor immunity against LLC.

## Discussion

Solid tumors have strong resistance and plasticity [31, 32], compromising the efficiency of many treatment methods [33, 34], and TRT is not an exception. Although β-radionuclides, particularly the clinically prevalent ^131^I, are suitable for solid tumor treatment due to their longer range and cross-fire effect, their antitumor efficiency is hampered by tumor hypoxia since ^131^I-represented β-radionuclides therapy needs O_2_ participation when producing ROS. Given the challenges posed by the immunosuppressive TME, incorporating additional therapeutic mechanisms to stimulate robust antitumor immunity represents a promising strategy to overcome the limitations of TRT monotherapy. However, such approaches are still constrained by tumor-intrinsic metabolic reprogramming, including aberrant lipid metabolism, which contributes to therapeutic resistance and tumor plasticity.

**Fig. 8:**
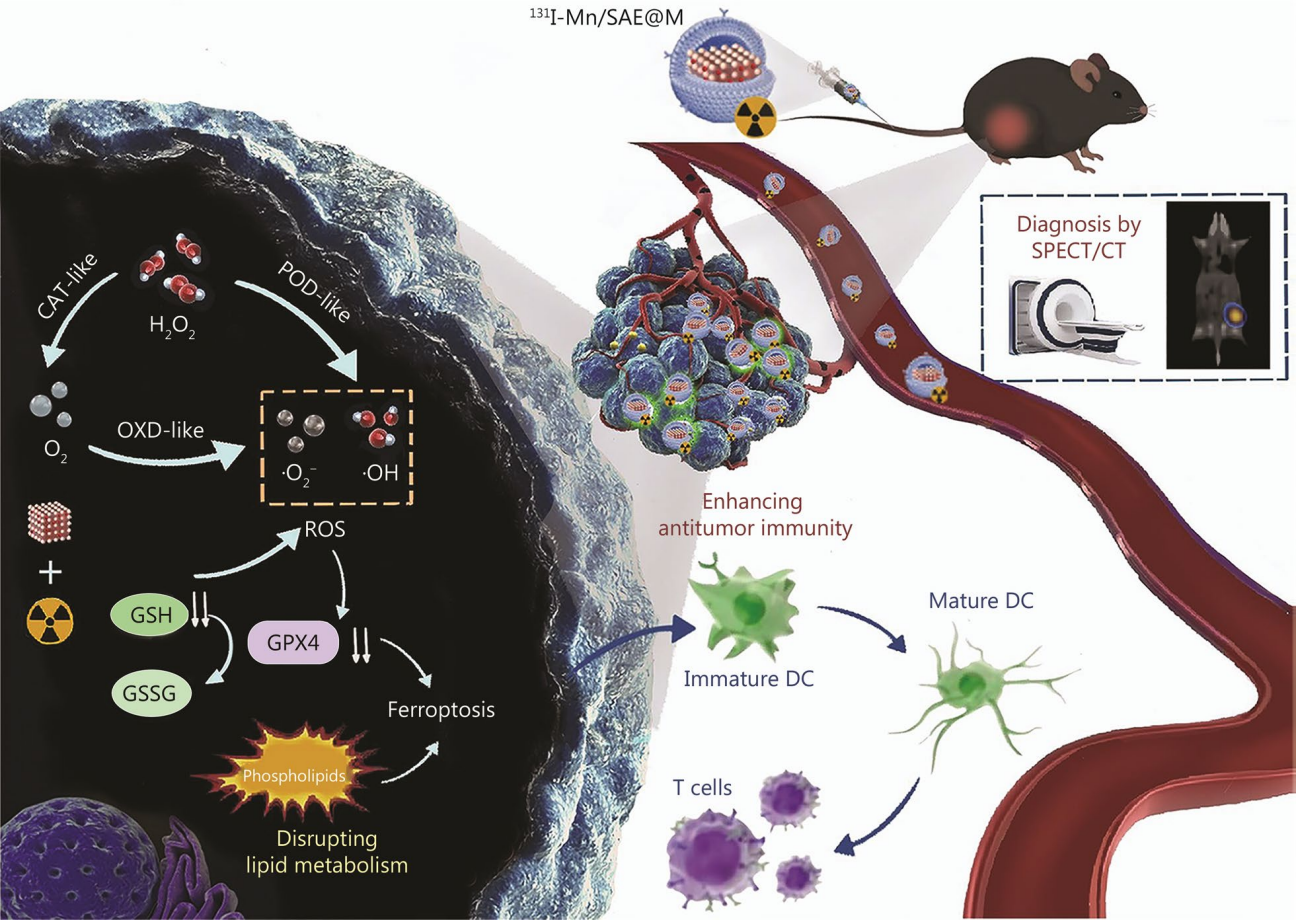
Schematic of the action principles of ^131^I-Mn/SAE@M in disrupting tumor lipid metabolism and remodeling the tumor immune microenvironment. CAT catalase, DC dendritic cell, GPX4 glutathione peroxidase 4, GSH glutathione, GSSS Oxidized glutathione, OXD oxidase, POD peroxidase, ROS reactive oxygen species, ^131^I-Mn/SAE@M iodine-131-membrane-coated manganese single-atom nanozymes, SPECT/CT single-photon emission computed tomography/computed tomography

Herein, the O_2_ release by the Mn/SAE-unlocked multienzymatic catalysis is capable of relieving the hypoxic microenvironment, preliminarily removing the resistance to ROS therapy [35]. Furthermore, it has been documented that lipid metabolism disruption can induce ferroptosis [36–40], and both GPX4-correlated ferroptosis promotion and lipid metabolism disruption favorably mitigate immunosuppressive TME and enhance immune responses [40–45]. More significantly, ferroptosis and lipid reprogramming have been validated to correlate with cGAS-STING pathway activation [46–50], which plays a central role in initiating innate immune responses through cytosolic DNA sensing [51–54]. Manganese itself has also been shown to enhance the sensitivity of the cGAS-STING pathway [55, 56]. Inspired by them, our engineered radiopharmaceutical ^131^I-Mn/SAE@M is predicated on multienzyme-mimicking cascade reactions to disrupt the intratumoral lipid metabolic homeostasis, activate the cGAS-STING pathway, dampen immunosuppressive microenvironment, induce ferroptosis, and directly achieve combined therapy with TRT. Contributed by them, such ^131^I-Mn/SAE@M radiopharmaceuticals potentiated robust antitumor immunological responses to repress the first LLC and second LLC through the activated immune memory effects. Importantly, we also shed light on the relationship between lipid metabolism homeostasis and tumor immunity at the proteomic and genomic levels. Targeting lipid metabolism to activate the cGAS-STING pathway and ferroptosis for potentiating antitumor immunity is identified as the mechanism of SAE-combined TRT (Fig. 8).

## Conclusions

In summary, we successfully developed a novel radiopharmaceutical ^131^I-Mn/SAE@M with multienzyme-mimicking catalytic activity, which can effectively inhibit tumor growth by disturbing the intracellular lipid metabolic homeostasis. Through cascade catalytic reactions (CAT-, POD-, OXD-, and GSHOx-like), ^131^I-Mn/SAE@M reversed tumor hypoxia, enhanced ROS accumulation, and induced oxidative damage. Most importantly, above distinctive characteristics favored ^131^I-Mn/SAE@M to disrupt lipid metabolic homeostasis through inhibiting cellular phospholipid synthesis, activate cGAS-STING pathway, induce ferroptosis, and, thereby, significantly reprogram hypoxic and immunosuppressive TME to activate and enhance the antitumor immunity. Contributed by them, the first LLC was repressed, and coincidentally, the second tumor was also inhibited through the activated immune memory effects. Therefore, the engineered radiopharmaceutical ^131^I-Mn/SAE@M, predicated on multienzyme-mimicking cascade reactions to disrupt the intratumoral lipid metabolic homeostasis, exhibited potent antitumor immunological responses. Overall, this study is expected to provide a new direction for the development and clinical translation of radiopharmaceuticals. The action rationales based on lipid metabolism homeostasis disruption and multienzyme-mimicking cascade reactions presented a distinctive antitumor strategy to synergize with TRT-based antitumor immunity.

## Supplementary Information

**Additional file 1.** Methods and materials. **Fig. S1** The EDS mapping of C, N, and Mn of the prepared ZIF-8 NCs. **Fig. S2** XPS spectra of Mn/SAE. **Fig. S3** Characterization of Mn/SAE@M. **Fig. S4** TEM image of Mn/SAE@M. **Fig. S5** DLS and zeta potential of Mn/SAE and Mn/SAE@M. **Fig. S6** EDS mapping of C, N, O, Mn, and I of the prepared I-Mn/SAE@M. **Fig. S7** Drug release profile of ¹³¹I-Mn/SAE@M under different pH conditions (7.4, 6.5, 5.5, 4, and 3) at various time points (1, 3, 6, 12, and 24 h). **Fig. S8 **The O^2-^generation efficiency measured by bleaching the DPBF absorbance at 420 nm at different times.** Fig. S9** Images and quantitative data for OXD-like activity of the Mn/SAE@M using TMB as an indicator. **Fig. S10** Images and quantitative data for POD-like activity of the Mn/SAE@M in the presence of H2O2 using TMB as an indicator. **Fig. S11** POD-like activity of the Mn/SAE@M using MB as an indicator. **Fig. S12** Representative images and quantification of LLC cells that experienced different treatments after DCFH-DA staining. **Fig. S13** Intracellular ROS generation induced by ^131^I-Mn/SAE@M, detected by DHE staining for ·O^2-^, HPF staining for ·OH, MitoPY1 staining for H_2_O_2_. **Fig. S14** Representative images of O_2_ generation in vitro. **Fig. S15** Cytotoxicity of ^131^I, Mn/SAE, ^131^I-Mn/SAE@M on tumor cells by using co-staining with Calcein-AM and PI. **Fig. S16** Quantification of GPX4 protein levels after different treatments. **Fig. S17** The ATP levels in LLC cells after different treatments. **Fig. S18** Visualization of JC-1 monomer and JC-1 aggregate after different treatments. **Fig. S19** Biodistribution of ^131^I-Mn/SAE@M in LLC tumor-bearing mice at 6, 24, or 48 post-intravenous injection. **Fig. S20** Individual tumor growth curves of LLC tumor-bearing mice in corresponding treatment groups (*n* = 6). **Fig. S21** Representative histological examinations of the tumor from each group with hematoxylin and eosin staining.** Fig. S22** Representative histological examinations of the main organs from each group with hematoxylin and eosin staining. **Fig. S23** Hemanalysis was performed on blood withdrawn from LLC tumor-bearing mice in corresponding treatment groups at the terminal of the study (*n* = 3). **Fig. S24** Differences in the contents of each lipid subclass among different treatment groups.** Fig. S25** Principal component analysis (PCA) score plot. **Fig. S26** Hierarchical clustering analysis based on significant difference in lipids. **Fig. S27** Differences in the content of lipid molecules with various chain lengths under each class. **Fig. S28** Differences in the content of lipid molecules with various degrees of carbon saturation under each class using one-way ANOVA without post hoc test. **Fig. S29** Immunogenic cell death induced by ^131^I-Mn/SAE@M. **Fig. S30** Gating strategies. **Fig. S31** Expression levels of serum cytokines, including IL-10, TGF-β, and IL-12 in the mice after different treatments. **Fig. S32** Immune response activation and lipid metabolism disruption after treatment with ^131^I-Mn/SAE@M. **Fig. S33** Gating strategies. **Fig. S34** Individual tumor growth curves of LLC tumor-bearing mice in the corresponding treatment groups (*n* = 5). **Fig. S35 **Gating strategies.

**Additional file 2.** **Video S1** The CAT-like activity of the Mn/SAE@M.

## Data Availability

All data can be obtained from the corresponding authors upon reasonable request.

## Abbreviations

ATP: Adenosine triphosphate
CAT: Catalase
CCK-8: Cell counting kit-8
CLSM: Confocal laser scanning microscopy
CRT: Calreticulin
DAMPs: Damage-associated molecular patterns
DAPI: 4'-6-Diamidino-2-phenylindole
DCs: Dendritic cells
DCFH-DA: 2'-7'-Dichlorodihydrofluorescein diacetate
DHE: Dihydroethidium
DMEM: Dulbecco’s modified Eagle medium
DMPO: Dimethylpyridine nitroxide
DTNB: 5,5'-Dithiobis-(2-nitrobenzoic acid)
EDS: Energy-dispersive X-ray spectroscopy
ESR: Electron spin resonance
FBS: Fetal bovine serum
FTIR: Fourier transform infrared spectroscopy
cGAS: Cyclic GMP-AMP synthase
GPX4: Glutathione peroxidase 4
GSH: Glutathione
GSHOx: Glutathione oxidase
HAADF STEM: High-angle annular dark-field scanning transmission electron microscopy
HIF-1α: Hypoxia-inducible factor-1α
HMGB1: High mobility group box 1
HPF: Hydroxyphenyl fluorescein
ICD: Immunogenic cell death
ICP-MS: Inductively coupled plasma-mass spectrometry
IFN-γ: Interferon-gamma
IRF3: Interferon regulatory factor 3
^131^I: Iodine-131
LLC: Lewis lung carcinoma
MB: Methylene blue
MDA: Malondialdehyde
MDSCs: Marrow-derived suppression cells
MFI: Mean fluorescence intensity
Mn/SAE: Manganese single-atom nanozymes
NSCLC: Non-small cell lung cancer
O_2_: Oxygen
OH: Hydroxyl radicals
OPLS-DA: Orthogonal partial least squares discriminant analysis
OXD: Oxidase
PBS: Phosphate buffered saline
PC: Phosphatidylcholine
PCA: Principal component analysis
PE: Phosphatidylethanolamine
PG: Phosphatidylglycerol
POD: Peroxidase
PI: Propidium iodide
PI: Phosphatidylinositol
PS: Phosphatidylserine
ROS: Reactive oxygen species
SAE: Single-atom nanoenzymes
SDS-PAGE: Sodium dodecyl sulfate–polyacrylamide gel electrophoresis
SEM: Scanning electron microscopy
SPECT/CT: Single-photon emission computed tomography/Computed tomography
STING: Stimulator of interferon genes
TA: Tannic acid
TAMs: Tumor-associated macrophages
TBK1: TANK-binding kinase 1
T_EM_: Immune effector memory T cells
TEM: Transmission electron microscopy
TG: Triglyceride
TMB: 3,3',5,5'-Tetramethylbenzidine
TME: Tumor microenvironment
TNF-α: Tumor necrosis factor-α
Tregs: Regulatory T cells
TRT: Targeted radionuclide therapy
VIP: Variable importance for the projection
XPS: X-ray photoelectron spectroscopy
XRD: X-ray powder diffraction
ZIF-8 HNCs: Zeolitic imidazolate frame-8 hollow nanocubes
