# Supplementary material for: Camouflaged membrane-bridged radionuclide/Mn single-atom enzymes target lipid metabolism disruption to evoke antitumor immunity
Source: Mil Med Res. 2025 Sep 19;12:59. doi: 10.1186/s40779-025-00647-7 (PMC12447611; doi:10.1186/s40779-025-00647-7)
Supplement: Supplementary file 1 — Additional file 1. Methods and materials. Fig. S1 The EDS mapping of C, N, and Mn of the prepared ZIF-8 NCs. Fig. S2 XPS spectra of Mn/SAE. Fig. S3 Characterization of Mn/SAE@M. Fig. S4 TEM image of Mn/SAE@M. Fig. S5 DLS and zeta potential of Mn/SAE and Mn/SAE@M. Fig. S6 EDS mapping of C, N, O, Mn, and I of the prepared I-Mn/SAE@M. Fig. S7 Drug release profile of ¹³¹I-Mn/SAE@M under different pH conditions (7.4, 6.5, 5.5, 4, and 3) at various time points (1, 3, 6, 12, and 24 h). Fig. S8 The O2-generation efficiency measured by bleaching the DPBF absorbance at 420 nm at different times. Fig. S9 Images and quantitative data for OXD-like activity of the Mn/SAE@M using TMB as an indicator. Fig. S10 Images and quantitative data for POD-like activity of the Mn/SAE@M in the presence of H2O2 using TMB as an indicator. Fig. S11 POD-like activity of the Mn/SAE@M using MB as an indicator. Fig. S12 Representative images and quantification of LLC cells that experienced different treatments after DCFH-DA staining. Fig. S13 Intracellular ROS generation induced by 131I-Mn/SAE@M, detected by DHE staining for ·O2-, HPF staining for ·OH, MitoPY1 staining for H2O2. Fig. S14 Representative images of O2 generation in vitro. Fig. S15 Cytotoxicity of 131I, Mn/SAE, 131I-Mn/SAE@M on tumor cells by using co-staining with Calcein-AM and PI. Fig. S16 Quantification of GPX4 protein levels after different treatments. Fig. S17 The ATP levels in LLC cells after different treatments. Fig. S18 Visualization of JC-1 monomer and JC-1 aggregate after different treatments. Fig. S19 Biodistribution of 131I-Mn/SAE@M in LLC tumor-bearing mice at 6, 24, or 48 post-intravenous injection. Fig. S20 Individual tumor growth curves of LLC tumor-bearing mice in corresponding treatment groups (n = 6). Fig. S21 Representative histological examinations of the tumor from each group with hematoxylin and eosin staining. Fig. S22 Representative histological examinations of the main organs from each group with hematoxylin [file 40779_2025_647_MOESM1_ESM.pdf]

Materials and methods

Materials

Manganese (II) chloride tetrahydrate ($MnCl_2·4H_2O$) and dimethyl sulfoxide (DMSO) were purchased from Sigma-Aldrich Chemical Co., Ltd. (St. Louis, MO, USA). 3,3',5,5'-tetramethylbenzidine (TMB), methylene blue (MB), and sodium pyrosulfite ($Na_2S_2O_5$) were provided by Shanghai Macklin Biochemical Technology Co., Ltd. Rhodamine B was acquired from Solarbio (Beijing, China). Chloramine T and potassium iodide (KI) were purchased from RHAWN (Shanghai, China). $Na^{131}I$ solution was supplied by Shanghai Xinke Pharmaceutical Co., Ltd. Phosphate-buffered saline (PBS), fetal bovine serum (FBS), Dulbecco's modified Eagle medium (DMEM), and a cell membrane protein extraction kit were purchased from Biosharp (Beijing, China). Calcein/propidium iodide (PI) cell viability/cytotoxicity assay kit, adenosine 5'-triphosphate (ATP) assay kit, reduced glutathione (GSH) JC-1 assay kit, and oxidized glutathione disulfide (GSSG) assay kit were obtained from Beyotime Biotechnology Co., Ltd. (Shanghai, China). The Annexin V-Fluorescein isothiocyanate (FITC) apoptosis assay kit and HY-126793 2′,7′-dichlorofluorescein diacetate (DCFH-DA) kit were purchased from MedChemExpress Co., Ltd. (NJ, USA). BBoxiProbe® O96 staining kit was purchased from BestBio Co., Ltd. (Nanjing, China). All antibodies used for the Western blotting assay were provided by Abmart Shanghai Co., Ltd. Ultrapure water (Milli-Q, Millipore, Bedford, MA, USA) was used in the experiment. MS-grade methanol, MS-grade acetonitrile, and HPLC-grade 2-propanol were purchased from Thermo Fisher (MA, USA). HPLC-grade formic acid and HPLC-grade ammonium formate were purchased from Sigma-Aldrich (St. Louis, USA).

Instruments

The hydrodynamic diameter and Zeta potential of the nanoparticles were analyzed by the Malvern Zetasizer Nano ZS (Malvern Panalytical, Malvern, UK). Scanning electron microscopy (SEM) images were taken by ZEISS Sigma 300 (Carl Zeiss, Oberkochen, Germany). Transmission electron microscopy (TEM) images were taken by FEI-Tecnai12 (Hillsboro, OR, USA). An Agilent 7800 (Agilent Technologies, Santa Clara, CA, USA) inductively coupled plasma-mass spectrometer (ICP-MS) was used to detect cellular uptake of materials. X-ray photoelectron spectroscopy (XPS) was collected by the K-Alpha X-ray Photoelectron Spectrometer System (Thermo Scientific, USA). The X-ray diffraction (XRD) pattern of the material was recorded by the Bruker D2 Phaser XRD

instrument (Bruker, Billerica, MA, USA). Energy dispersive X-ray spectroscopy (EDS) mapping elemental analysis was performed at JEM-F200 (URP) (JEOL Ltd., Tokyo, Japan). Fourier-transform infrared spectroscopy (FTIR) was collected by a Thermo Scientific instrument (Thermo Fisher Scientific, Waltham, MA, USA). Fluorescence imaging was performed by Carl Zeiss LSM900 confocal microscope (Carl Zeiss, Oberkochen, Germany) and Nikon inverted fluorescence microscope (Nikon, Tokyo, Japan). Absorbance and chemiluminescence were measured using the SpectraMax iD5 Multi-Mode Microplate Readers (Molecular Devices, USA). The polyvinylidene fluoride (PVDF) membrane was visualized by a chemiluminescence scanner (Tanon, Shanghai, China). Flow cytometry was performed on a BD Fortessa flow cytometer (BD Biosciences, San Jose, CA, USA). Imaging of ^{131}I was performed using single-photon emission computed tomography/computed tomography (SPECT/CT) (GE Discovery 670, USA).

Material characterization

Characterization of Mn/SAE and Mn/SAE@M

The size distribution and surface zeta potential of Mn/SAE and Mn/SAE@M were evaluated by dynamic light scattering (DLS). The morphology of Mn/SAE and Mn/SAE@M was characterized by SEM and TEM. The diffraction pattern of Mn/SAE was obtained by XRD. The chemical composition and elemental valence states were analyzed by XPS. EDS mapping was used to determine the elemental distribution of Mn/SAE@M. The functional groups and chemical bonds were analyzed by FTIR.

Identification of proteins in cancer cell membranes and Mn/SAE@M nanoparticles

Sodium dodecyl sulfate-polyacrylamide gel electrophoresis (SDS-PAGE) was used to analyze the types and contents of proteins in Lewis lung carcinoma (LLC) cell membranes, Mn/SAE@M, and Mn/SAE. The SDS-PAGE protein up-sampling buffer was added to LLC cell membrane proteins and nanoparticles, and these mixtures were heated at 100 °C for 5 min to fully denature the proteins. Prepared samples of each protein (20 μg) were added to a 10% SDS-PAGE gel for electrophoresis and then stained with Coomassie blue for 1 h. Decolorization was performed until the background was clean and clear for imaging.

Catalase (CAT)-like activity assay of Mn/SAE@M

The CAT-like activity of Mn/SAE@M was evaluated at room temperature by observing its ability to

induce oxygen production. The CAT-like activity of Mn/SAE@M was evaluated by detecting dissolved O_2 at room temperature using a portable dissolved oxygen meter. The oxygen electrode was immersed in the test solution containing nanozymes (80 μg/ml) and H_2O_2 (10 mmol/L), and then the dissolved O_2 values were recorded. Dissolved O_2 was measured dynamically at 20-second intervals during a 10-minute monitoring period. Besides, H_2O_2 (2 ml, 5 mmol/L) was mixed with HAc-NaAc buffer (7 ml, 0.01 mol/L, pH = 5.0), and then Mn/SAE@M (2 ml, 2 mg/ml) was added. Another group to which only H_2O_2 and HAc-NaAc buffer were added served as a control. Then, we recorded the experimental phenomena by taking videos.

Oxidase (OXD)-like activity assay of Mn/SAE@M

The OXD-like activity of Mn/SAE@M was evaluated using TMB as a chromogenic substrate at room temperature. The absorbance of each solution post-reaction with Mn/SAE@M at 650 nm was recorded using a microplate reader.

The concentration dependency of the OXD-like activity of Mn/SAE@M was first evaluated. Different concentrations of Mn/SAE@M (0, 12.5, 25, 50, 75, and 100 μg/ml) were mixed with NaAc-HAc buffer (0.01 mmol/L, pH = 5.0), followed by the addition of 1 mmol/L TMB (dissolved in DMSO) to the mixture. The effect of TMB concentration on the detection of the OXD-like activity of Mn/SAE@M was conducted in NaAc-HAc buffer (0.01 mol/L, pH = 5.0). Different concentrations of TMB solution (0, 0.2, 0.4, 0.6, 0.8, and 1.0 mmol/L) were added, and upon the conversion of colorless TMB to blue oxidized TMB.

To investigate the effect of pH on the OXD-like activity of Mn/SAE@M, 100 μg/ml of Mn/SAE@M was mixed with NaAc-HAc buffer (0.01 mol/L, pH = 4.0, 5.0, or 6.0) or PBS (pH = 7.4), followed by the addition of 1 mmol/L TMB to the mixture.

Peroxidase (POD)-like activity assay of Mn/SAE@M

POD-like activity of Mn/SAE@M promoting ·OH generation was analyzed by a colorimetric reaction using TMB (dissolved in DMSO) as substrate at room temperature. After a certain time of reaction, the absorbance of the colored solution at 650 nm was measured by a microplate reader. The concentration dependence of the POD-like activity of Mn/SAE@M was first evaluated. Different concentrations of Mn/SAE@M (0, 12.5, 25, 50, 75, and 100 μg/ml) and H_2O_2 (2.5 mmol/L) were mixed with HAc-NaAc buffer (0.01 mol/L, pH = 5.0) before adding TMB (1 mmol/L) to the mixture. The assay was performed after incubation for 10 min at room temperature. The effect of pH on the

POD-like activity of Mn/SAE@M was then determined. Mn/SAE@M (100 μg/ml) and H_2O_2 (2.5 mmol/L) were mixed with HAc-NaAc buffer (0.01 mol/L, pH = 4.0, 5.0, and 6.0) or PBS buffer (pH = 7.4) before adding TMB (1 mmol/L) to the mixture. The assay for the effect of TMB concentration on Mn/SAE@M activity was performed in HAc-NaAc buffer (0.01 mol/L, pH = 5.0). Mn/SAE@M (100 μg/ml), H_2O_2 (2.5 mmol/L), and different amounts (0, 0.2, 0.4, 0.6, 0.8, and 1.0 mmol/L) of TMB solution were added.

The effect of H_2O_2 concentration on the POD-like activity of Mn/SAE@M was determined by using Mn/SAE@M (100 μg/ml) and TMB (1 mmol/L) dissolved in HAc-NaAc buffer (0.01 mol/L, pH = 5.0). Different amounts (0, 0.5, 1.0, 1.5, 2.0, and 2.5 mmol/L) of H_2O_2 were added.

The MB degradation serves as a paradigm for demonstrating POD-like catalysis that uses the same conjugation process. The reaction mixture was incubated at room temperature, and the absorbance of MB at 664 nm was recorded using a microplate reader to monitor the degradation process. The decrease in absorbance over time indicates the catalytic decomposition of MB, thereby reflecting the POD-like activity of Mn/SAE@M.

Glutathione oxidase (GSHOx)-like activity assay of Mn/SAE@M

The GSHOx-like activity of Mn/SAE@M was determined in PBS solution (pH = 8.0) via the colorimetric method using 5,5'-dithiobis-(2-nitrobenzoic acid) (DTNB) as chromophoric substrate. In detail, 80 μl of 1 mg/ml nanozyme solution and 50 μl of 10 mmol/L glutathione (GSH) solution were added to 0.87 ml PBS solution and mixed thoroughly. The mixture was kept at room temperature for 4 h. Then, 0.1 ml of the above solution was taken and diluted to 1 ml with PBS solution, which was followed by the addition of DTNB (20 μl, 20 mmol/L).

Cell experiments

In vitro cytotoxicity analysis

Cell viability was measured by the cell counting kit-8 (CCK-8) method. LLC cells were seeded into 96-well plates at a density of 5000 cells per well and cultured in neutral or acidic DMEM containing 10% FBS (100 μl) for 24 h. The old medium was removed, and the cells were treated with serum-free DMEM containing different concentrations of ^{131}I, Mn/SAE (with or without 100 μmol/L H_2O_2), or ^{131}I-Mn/SAE@M. After 24 h, the medium was sucked out, replaced with fresh DMEM, and CCK-8 solution (10 μl) was added to each well. Then the absorbance was measured at 450 nm by a microplate reader, and the cell survival rate was calculated.

Live/dead cell staining test

The in vitro efficacy of ^{131}I-Mn/SAE@M was further evaluated by live/dead staining. LLC cells were seeded into 6-well plates (3 × 10^5 cells per well) and incubated overnight. ^{131}I (300 μCi/ml), Mn/SAE (180 μg/ml), or ^{131}I-Mn/SAE@M (300 μCi/ml) were added for treatment, and the untreated group was used as a control. After 24 h, the cells were stained with Calcein-AM and PI. After incubation at 37 ℃ for 1 h, the staining effect was observed by imaging under a fluorescence microscope.

Apoptosis assay

LLC cells were seeded in 6-well plates (5 × 10^5 per well) and allowed to adhere and grow for 24 h. The old medium was then replaced with DMEM containing ^{131}I (500 μCi/ml), Mn/SAE (300 μg/ml), and ^{131}I-Mn/SAE@M (500 μCi/ml). After 14 h, cells treated differently were harvested and stained with Annexin V-FITC (5 μl) and PI (5 μl) according to the manufacturer's instructions. The apoptosis of each cell sample was analyzed by flow cytometry. The obtained data were processed by Flow Jo software.

Detection of intracellular GSH and GSSG levels

The ability of ^{131}I-Mn/SAE@M to deplete GSH was assessed using GSH and GSSG assay kits. LLC cells were seeded in 6-well plates (5 × 10^5 cells per well) and cultured overnight to make them adherent. The cells were then co-incubated with ^{131}I (500 μCi/ml), Mn/SAE (300 μg/ml), and ^{131}I-Mn/SAE@M (500 μCi/ml) for 24 h. The cells were washed once with PBS buffer and gathered by centrifugation. Protein removal reagent was added to the collected cells according to the instructions of the assay kit, vortexed thoroughly, and then the samples were rapidly freeze-thawed twice. The supernatant was taken after centrifugation (10,000× *g*) for 10 min at 4 °C. One portion of the supernatant was used for the determination of total GSH, and the other was used for the determination of the GSSG content after the removal of GSH. The absorbance at 412 nm was measured with a microplate reader, and total GSH and GSSG were quantified by plotting a standard curve. Reduced GSH was calculated by subtracting double the amount of GSSG from total GSH.

Measurement of intracellular ATP levels

LLC cells were seeded into 6-well plates and cultured in an incubator until the cells were completely adhered to the wall. The medium in each well was then replaced with fresh DMEM containing ^{131}I

(500 μCi/ml), Mn/SAE (300 μg/ml), and ^{131}I-Mn/SAE@M (500 μCi/ml), respectively. Twenty-four hours later, the LLC cells were lysed according to the manufacturer's instructions for the ATP assay kit, and the supernatant collected by centrifugation was added to the assay solution. The ATP concentration was determined by recording the chemiluminescence of the solution with a multi-mode microplate reader. To eliminate errors due to differences in protein amounts, the protein content of the samples was quantified according to the bicinchoninic acid (BCA) method, and then the ATP concentration was converted.

Measurement of O_2 generation in vitro

LLC cells (1×10^5 cells in 1 ml medium) were seeded in the confocal laser scanning microscopy (CLSM) vessels. Then, these vessels were co-cultured with different treatments: ^{131}I (500 μCi/ml), Mn/SAE (300 μg/ml), and ^{131}I-Mn/SAE@M (500 μCi/ml), respectively. Twenty-four hours later, cells were stained with BBoxiProbe® O96, indicated by a fluorescence emission shift from red (excitation at 456 nm/emission at 612 nm). O_2 generation was determined using confocal microscopy.

Measurement of mitochondrial membrane potential

LLC cells (1×10^5 cells in 1 ml medium) were seeded in the CLSM vessels. Then, these vessels were co-cultured with different treatments: ^{131}I (500 μCi/ml), Mn/SAE (300 μg/ml), and ^{131}I-Mn/SAE@M (500 μCi/ml), respectively. After 24 h, cells were stained with JC-1 (5 μmol/L) for 30 min at 37 °C. JC-1 dye accumulates in mitochondria in a potential-dependent manner, as evidenced by a fluorescence emission shift from green (excitation at 485 nm/emission at 516 nm) to red (excitation at 579 nm/emission at 599 nm). Confocal microscopy was used to determine the mitochondrial membrane potential.

Animal experiments

SPECT/CT imaging

To better observe the distribution and metabolism of ^{131}I-Mn/SAE@M in vivo, a SPECT/CT scanner (GE Discovery 670, USA) was used to acquire whole-body images of mice. When the tumor volume of tumor-bearing mice reached about 400 mm^3, ^{131}I or ^{131}I-Mn/SAE@M was injected into the tail vein (n = 3). The mice were anesthetized with 3% pentobarbital by intraperitoneal injection at different time points (6, 12, 24, 48, and 96 h) after administration, and then scanned for follow-up

imaging.

In vivo biodistribution of ^{131}I-Mn/SAE@M

When the tumor volume reached about 400 mm^3, the tumor-bearing mice were randomly divided into 3 groups (6, 24, 48 h; $n = 3$). Each mouse was injected with ^{131}I-Mn/SAE@M (500 μCi) via the tail vein. After the corresponding time point was reached, the mice were euthanized. Tumors, as well as selected tissues and organs (heart, liver, spleen, lung, kidney, stomach, small intestine, thyroid, brain, bone, muscle), together with blood, were collected and weighed individually. The radioactive counts were performed with a gamma counter. Results are expressed as a percentage of the injected dose per gram of tissue (%ID/g).

Histology and immunohistochemistry

After 14 d of treatment, major organs (heart, lung, liver, spleen, and kidney) and tumor tissues of mice were collected and used to make hematoxylin and eosin (H&E) stained pathological sections for histological analysis. The terminal deoxynucleotidyl transferase dUTP nick end labeling (TUNEL) assay was designed to assess the degree of apoptosis in tumor tissues. In addition, the variation of STING, phosphorylated STING (pSTING), phosphorylated TANK-binding kinase 1 (pTBK1), phosphorylated interferon regulatory factor 3 (pIRF3), and glutathione peroxidase 4 (GPX4) expression in different tumor tissues was examined by immunohistochemistry with the corresponding antibody.

Bioanalytical methods

Western blotting analysis

Changes in GPX4 expression were detected using the Western blotting method. Cell lysates were obtained by adding protease inhibitor-pretreated RIPA lysate to differentially treated LLC cells. The BCA method was used to quantify the concentration of total protein in the cell lysis products. Samples loading buffer was added to the protein, and the mixture was denatured in a boiling water bath at 100 °C for 5 min. After separation of the proteins by SDS-PAGE gel electrophoresis, the proteins were transferred to a PVDF membrane. Next, the PVDF membrane was closed with 5% skimmed milk powder for 90 min at room temperature and then incubated with GPX4 antibody (T56959, Abmart, Shanghai, China) and β-actin (T40104, Abmart, Shanghai, China) overnight at 4 °C. After washing the membranes with TBST buffer, incubation was continued with the secondary

antibody Goat Anti-Rabbit IgG horseradish POD (M21002, Abmart, Shanghai, China) or Goat Anti-Mouse IgG horseradish POD (M21001, Abmart, Shanghai, China) for 90 min at room temperature. Finally, the membranes were visualized by chemiluminescent scanning with chemiluminescent reagents. And the gray value of the bands was measured on ImageJ.

LC-MS/MS method for lipid analysis

Lipid extraction was performed utilizing the methyl tert-butyl ether (MTBE) method. In brief, samples were initially augmented with a precise quantity of internal lipid standards, followed by homogenization in 200 µl of water and 240 µl of methanol. Subsequently, 800 µl of MTBE was introduced to the mixture, which was then subjected to ultrasonication for 20 min at 4 °C, and thereafter allowed to rest for 30 min at room temperature. The mixture was centrifuged at 14,000× *g* for 15 min at 10 °C, and the upper phase containing the organic solvent was collected and evaporated to dryness under a stream of nitrogen. The lipid extracts were redissolved in 200 µl of a 90% isopropanol/acetonitrile mixture, centrifuged at 14,000× *g* for 15 min, and subsequently, a 3 µl aliquot of the sample was injected into the system.

For the LC separation, reverse-phase chromatography was employed using a CSH C_{18} column (1.7 µm, 2.1 mm × 100 mm, Waters). The mobile phase consisted of solvent A (acetonitrile-water, 6:4, v/v, with 0.1% formic acid and 0.1 mmol/L ammonium formate) and solvent B (acetonitrile-isopropanol, 1:9, v/v, with 0.1% formic acid and 0.1 mmol/L ammonium formate). The chromatographic run was initiated with 40% solvent B at a flow rate of 300 µl/min, maintained for 3.5 min, followed by a linear increase to 75% solvent B over 9.5 min, and then to 99% solvent B over 6 min, concluding with re-equilibration at 40% solvent B for 5 min.

Mass spectral analysis was conducted on a Q-Exactive Plus instrument, in both positive and negative ionization modes. Electrospray ionization (ESI) parameters were meticulously optimized and consistently applied across all measurements as follows. Source temperature was set at 300 °C; capillary temperature at 350 °C, the ion spray voltage was established at 3000 V, S-Lens RF Level at 50%, and the scan range was configured from *m/z* 200 to 1800. “LipidSearch” is an advanced search engine designed for the identification of lipid species through MS/MS matching. It encompasses over 30 lipid classes and boasts a comprehensive database of more than 1.5 million fragment ions. The mass tolerance for both precursor and fragment ions has been precisely set to 5 ppm.

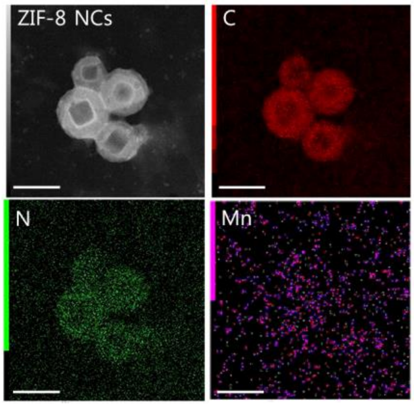

Fig. S1 The EDS mapping of C, N, and Mn of the prepared ZIF-8 NCs. Scale bar = 200 nm. EDS energy-dispersive X-ray spectroscopy, ZIF-8 zeolitic imidazolate frame-8, NCs nanocubes

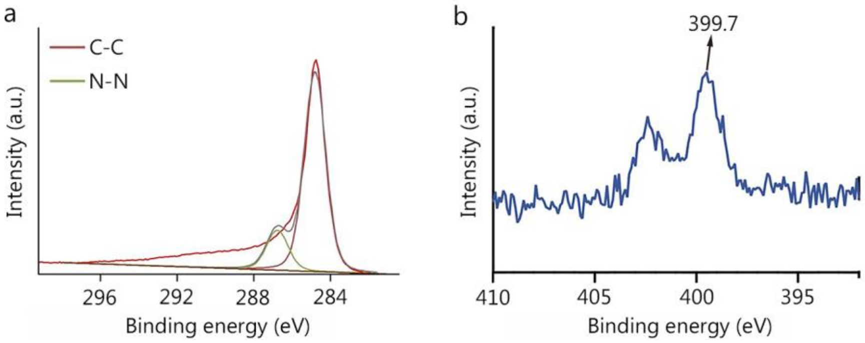

Fig. S2 XPS spectra of Mn/SAE. **a** C 1s. **b** N 1s. XPS X-ray photoelectron spectroscopy, Mn/SAE manganese-based single-atom nanozyme

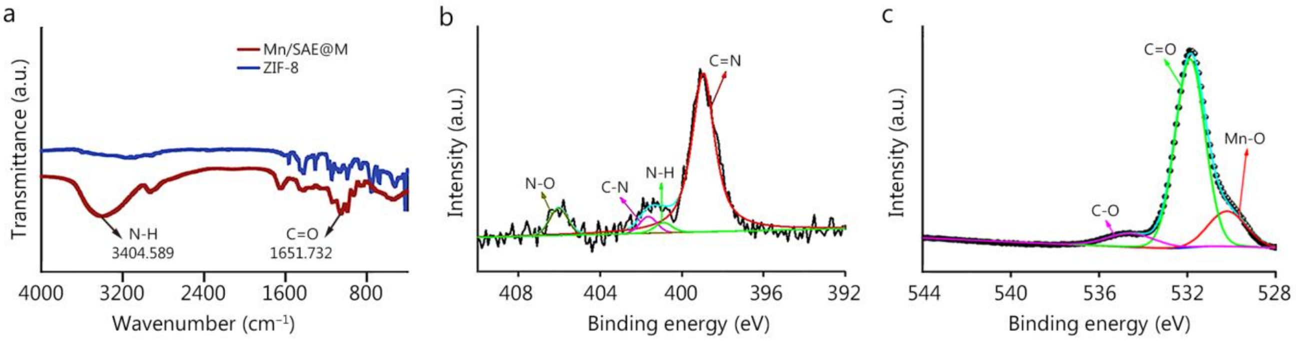

Fig. S3 Characterization of Mn/SAE@M. **a** FTIR spectroscopy of Mn/SAE@M and ZIF-8. **b** XPS spectra for N 1s of Mn/SAE@M. **c** XPS spectra for O 1s of Mn/SAE@M. Mn/SAE@M membrane-coated manganese single-atom nanozymes, FTIR fourier transform infrared spectroscopy, ZIF-8 zeolitic imidazolate frame-8, XPS X-ray photoelectron spectroscopy

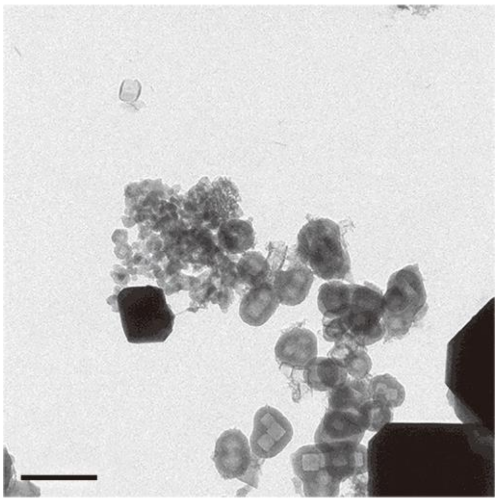

Fig. S4 TEM image of Mn/SAE@M. Scale bar = 0.5 μm. TEM transmission electron microscopy, Mn/SAE@M membrane-coated manganese single-atom nanozymes

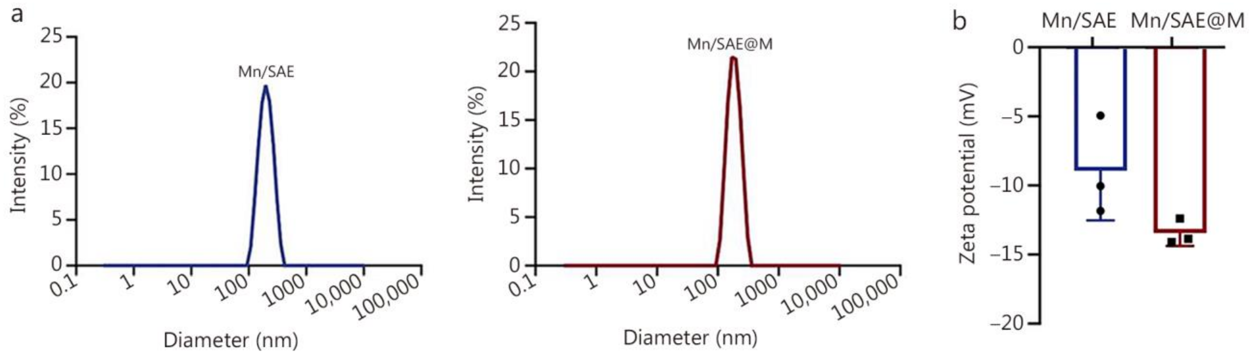

Fig. S5 DLS and zeta potential of Mn/SAE and Mn/SAE@M. **a** DLS of Mn/SAE and Mn/SAE@M. **b** Zeta potential of Mn/SAE and Mn/SAE@M. Data are expressed as mean ± SD (n = 3). SD standard deviation, DLS dynamic light scattering, Mn/SAE manganese-based single-atom nanozyme, Mn/SAE@M membrane-coated manganese single-atom nanozymes

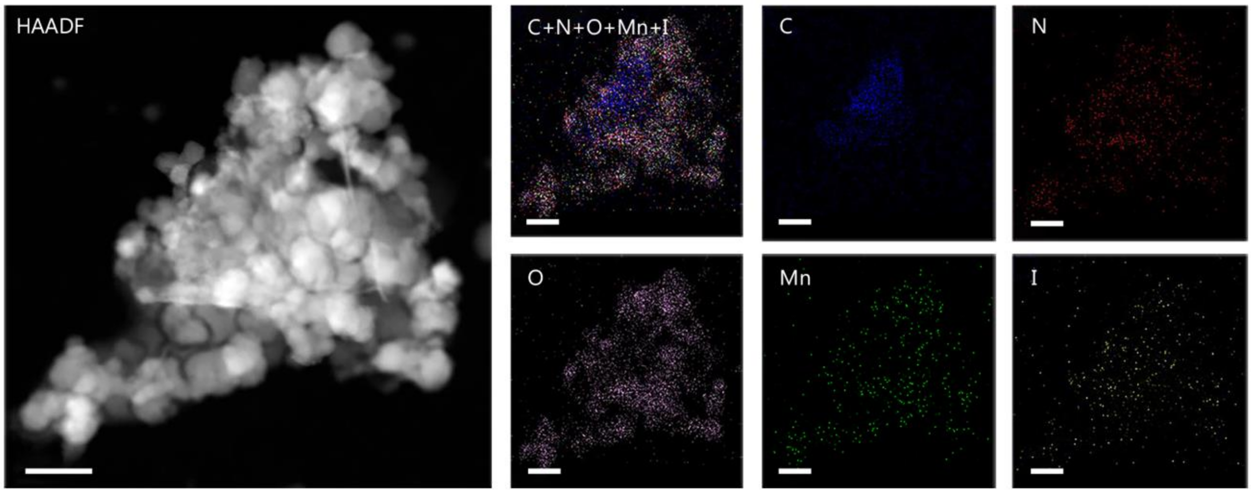

Fig. S6 EDS mapping of C, N, O, Mn, and I of the prepared ^{131}I-Mn/SAE@M. Scale bar = 200 μm. EDS energy dispersive X-ray spectroscopy, ^{131}I-Mn/SAE@M iodine-131-membrane-coated manganese single-atom nanozymes, HAADF high-angle annular dark-field

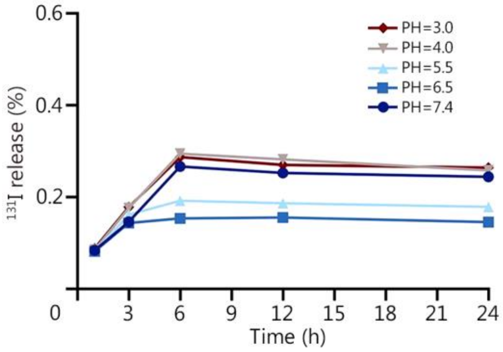

Fig. S7 Drug release profile of ^{131}I-Mn/SAE@M under different pH conditions (3.0, 4.0, 5.5, 6.5, and 7.4) at various time points (1, 3, 6, 12, and 24 h). Data are expressed as mean ± SD (*n* = 3). SD standard deviation, ^{131}I-Mn/SAE@M iodine-131-membrane-coated manganese single-atom nanozymes

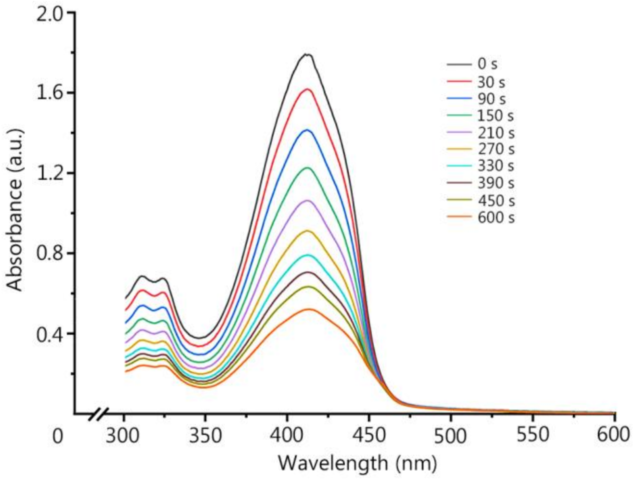

Fig. S8 The O_2^- generation efficiency measured by bleaching the DPBF absorbance at 420 nm at different times. Assays were performed on Mn/SAE@M (50 µg/ml Mn/SAE@M) with 2 mmol/L H_2O_2 and 30 µg/ml DPBF in acetic acid-sodium acetate buffer (pH = 6.0). DPBF 1,3-diphenylisobenzofuran, Mn/SAE@M membrane-coated manganese single-atom nanozymes

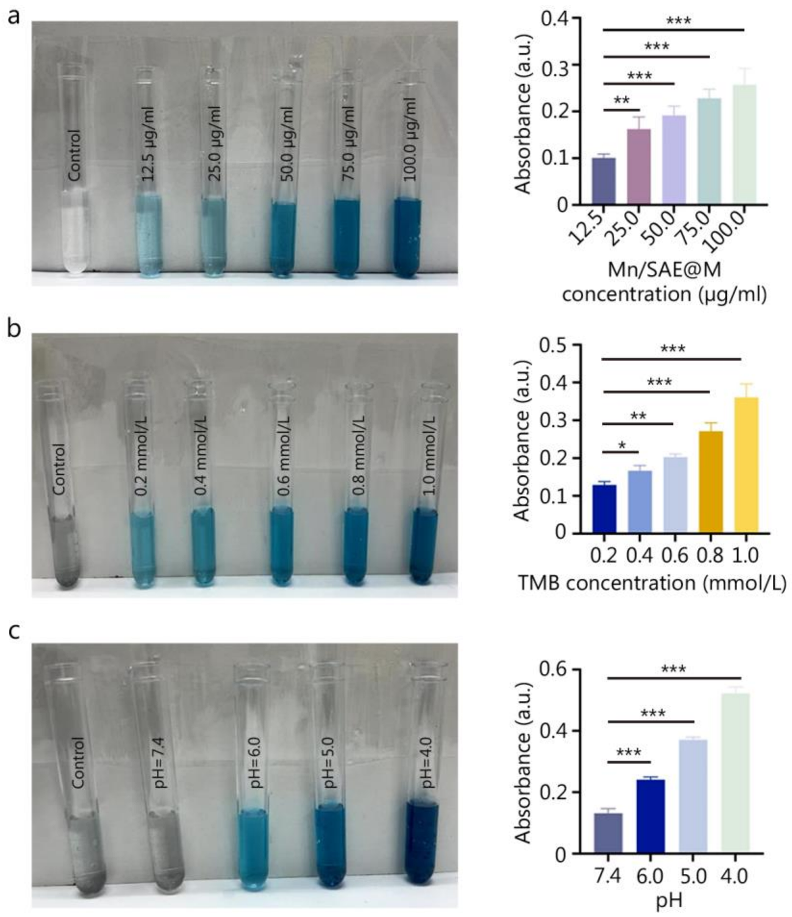

Fig. S9 Images and quantitative data for OXD-like activity of the Mn/SAE@M using TMB as an indicator. **a** TMB assay for measuring OXD-like activity of the Mn/SAE@M at different Mn/SAE concentrations (1.0 mmol/L TMB, pH = 5.0). **b** TMB assay for measuring OXD-like activity of the Mn/SAE@M at different concentrations of TMB (100 μg/ml Mn/SAE@M, pH = 5.0). **c** TMB assay for measuring OXD-like activity of the Mn/SAE@M at different pH (100 μg/ml Mn/SAE@M, 1.0 mmol/L TMB). Data are expressed as mean ± SD (n = 3). $^{*}P < 0.05$, $^{**}P < 0.01$, $^{***}P < 0.001$. SD standard deviation, OXD oxidase, Mn/SAE@M membrane-coated manganese single-atom nanozymes, TMB 3,3',5,5'-tetramethylbenzidine

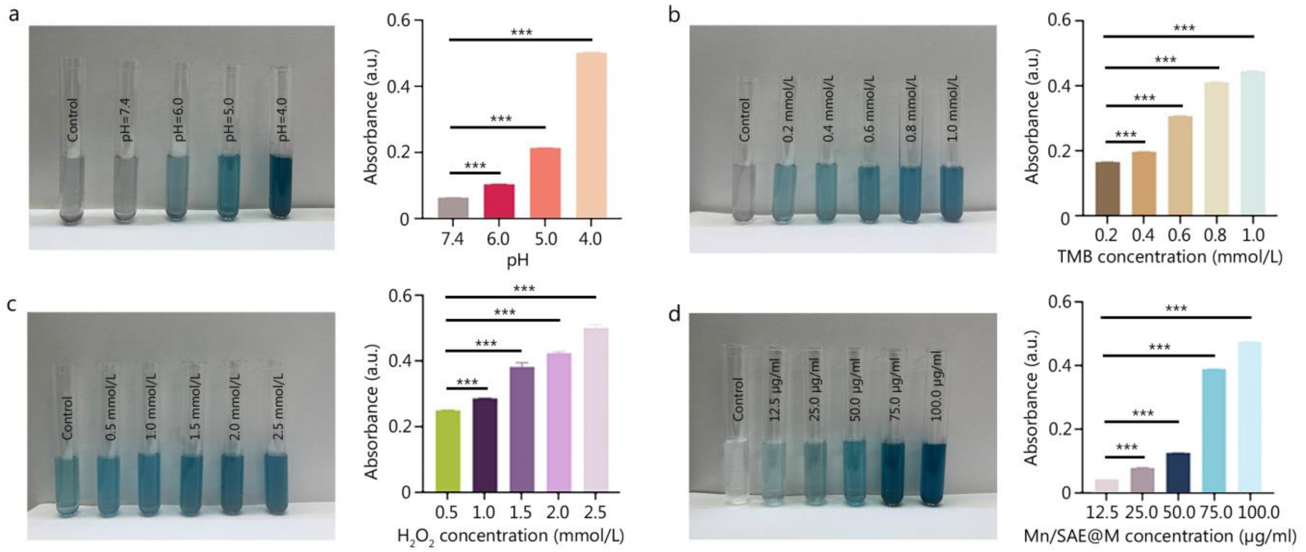

Fig. S10 Images and quantitative data for POD-like activity of the Mn/SAE@M in the presence of H_2O_2 using TMB as an indicator. **a** TMB assay for measuring POD-like activity of the Mn/SAE@M at the different pH (100 μg/ml Mn/SAE@M, 1.0 mmol/L TMB, 2.5 mmol/L H_2O_2). **b** TMB assay for measuring POD-like activity of the Mn/SAE@M at different concentrations of TMB (100 μg/ml Mn/SAE@M, 2.5 mmol/L H_2O_2; pH = 5.0). **c** TMB assay for measuring POD-like activity of the Mn/SAE@M at different H_2O_2 concentrations (100 μg/ml Mn/SAE@M, 1.0 mmol/L TMB; pH = 5.0). **d** TMB assay for measuring POD-like activity of the Mn/SAE@M at different Mn/SAE concentrations (1.0 mmol/L TMB, 2.5 mmol/L H_2O_2; pH = 5.0). Data are expressed as mean ± SD (*n* = 3). $^{***}P < 0.001$. SD standard deviation, POD peroxidase, Mn/SAE@M membrane-coated manganese single-atom nanozymes, TMB 3,3',5,5'-tetramethylbenzidine

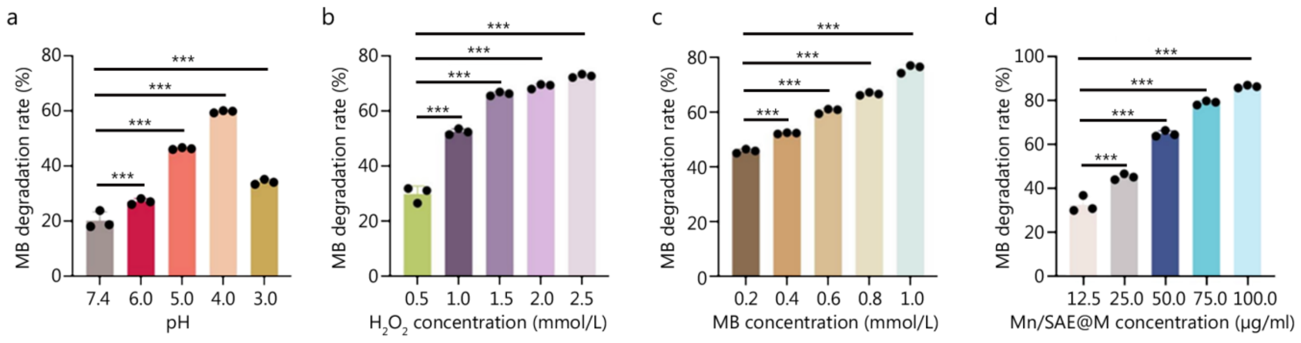

Fig. S11 POD-like activity of the Mn/SAE@M using MB as an indicator. **a** MB assay for measuring POD-like activity at different pH levels (100 μg/ml Mn/SAE@M, 1.0 mmol/L MB, 2.5 mmol/L H_2O_2). **b** MB assay for measuring POD-like activity at different H_2O_2 concentrations (100 μg/ml Mn/SAE@M, 1.0 mmol/L MB, pH=5.0). **c** MB assay for measuring POD-like activity at different concentrations of MB (100 μg/ml Mn/SAE@M, 2.5 mmol/L H_2O_2, pH = 5.0). **d** MB assay for measuring POD-like activity at different Mn/SAE@M concentrations (1.0 mmol/L MB, pH = 5.0, 2.5 mmol/L H_2O_2). Data are expressed as mean ± SD (*n* = 3). $^{***}P < 0.001$. SD standard deviation, POD peroxidase, Mn/SAE@M membrane-coated manganese single-atom nanozymes, MB methylene blue

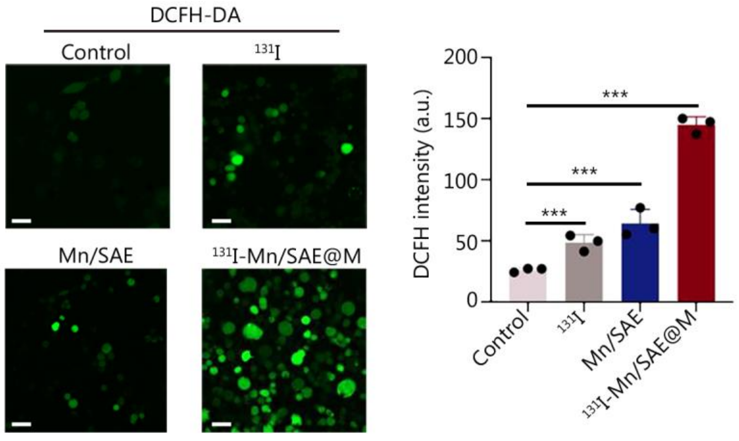

Fig. S12 Representative images and quantification of LLC cells that experienced different treatments after DCFH-DA staining. Scale bar = 20 μm. Data are expressed as mean ± SD (*n* = 3). $^{***}P < 0.001$. SD standard deviation, LLC Lewis lung carcinoma, DCFH-DA 2'-7'-dichlorodihydrofluorescein diacetate, ^{131}I iodine-131, Mn/SAE manganese-based single-atom nanozyme, ^{131}I-Mn/SAE@M iodine-131-membrane-coated manganese single-atom nanozymes

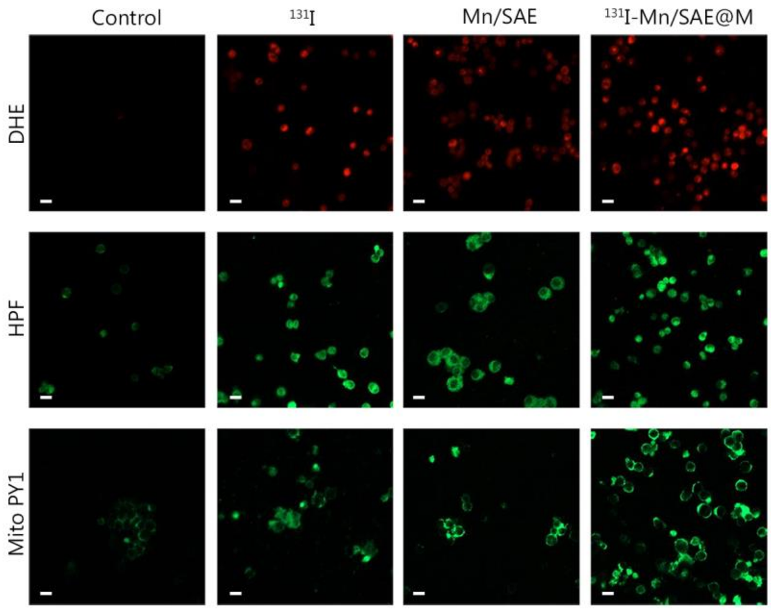

Fig. S13 Intracellular ROS generation induced by ^{131}I-Mn/SAE@M, detected by DHE staining for $\cdot O_2^-$, HPF staining for ·OH, MitoPY1 staining for H_2O_2. Scale bar = 20 μm. ROS reactive oxygen species, ^{131}I iodine-131, Mn/SAE manganese-based single-atom nanozyme, ^{131}I-Mn/SAE@M iodine-131-membrane-coated manganese single-atom nanozymes

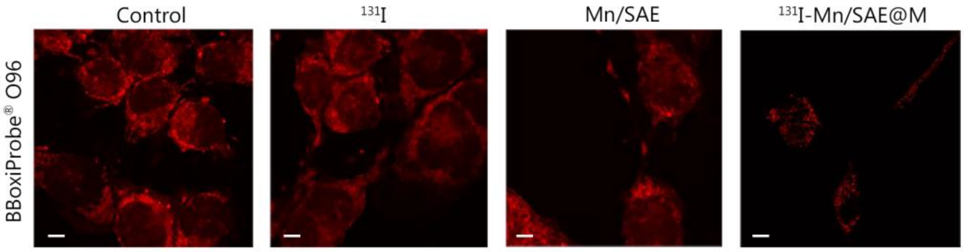

Fig. S14 Representative images of O_2 generation in vitro. Scale bar = 5 μm. ^{131}I iodine-131, Mn/SAE manganese-based single-atom nanozyme, ^{131}I-Mn/SAE@M iodine-131-membrane-coated manganese single-atom nanozymes

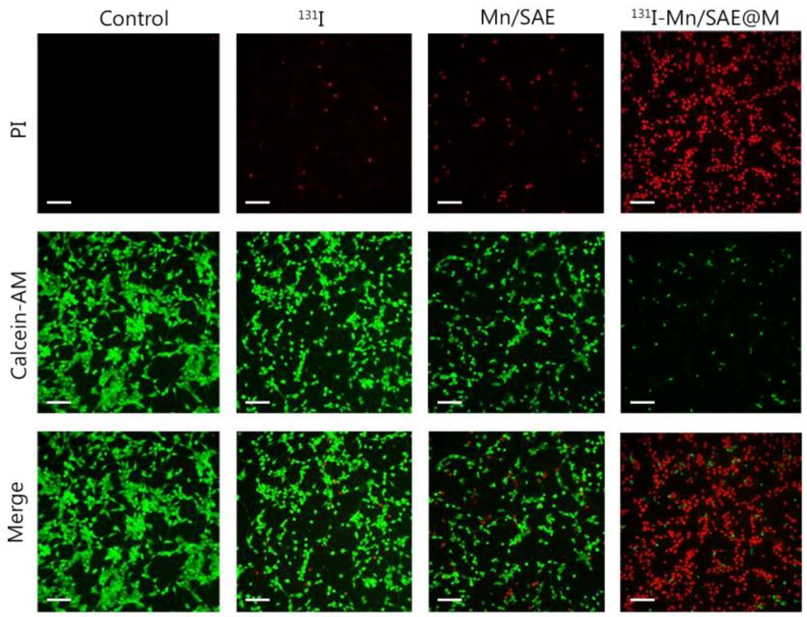

Fig. S15 Cytotoxicity of ^{131}I, Mn/SAE, and ^{131}I-Mn/SAE@M on tumor cells by using co-staining with PI and Calcein-AM. Scale bar = 20 μm. ^{131}I iodine-131, Mn/SAE manganese-based single-atom nanozyme, ^{131}I-Mn/SAE@M iodine-131-membrane-coated manganese single-atom nanozymes, PI propidium iodide, Calcein-AM calcein acetoxymethyl ester

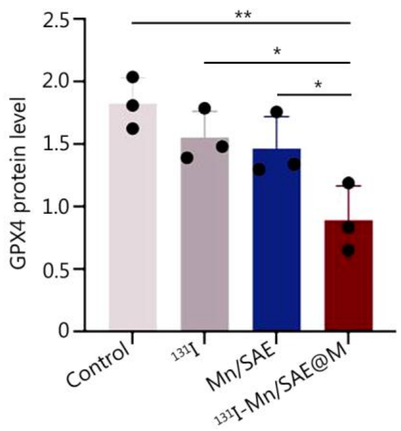

Fig. S16 Quantification of GPX4 protein levels after different treatments. Data are expressed as mean ± SD (n = 3). $^{*}P < 0.05$, $^{**}P < 0.01$. GPX4 glutathione peroxidase 4, SD standard deviation, ^{131}I iodine-131, Mn/SAE manganese-based single-atom nanozyme, ^{131}I-Mn/SAE@M iodine-131-membrane-coated manganese single-atom nanozymes

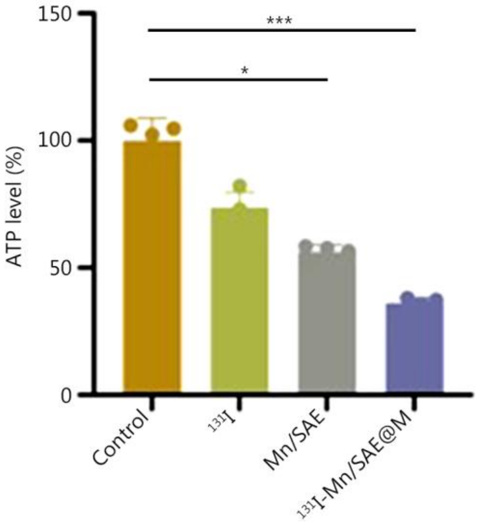

Fig. S17 The ATP levels in LLC cells after different treatments. Data are expressed as mean ± SD (*n* = 3). $^{*}P < 0.05$, $^{***}P < 0.001$. ATP adenosine triphosphate, LLC Lewis lung carcinoma, SD standard deviation, ^{131}I iodine-131, Mn/SAE manganese-based single-atom nanozyme, ^{131}I-Mn/SAE@M iodine-131-membrane-coated manganese single-atom nanozymes

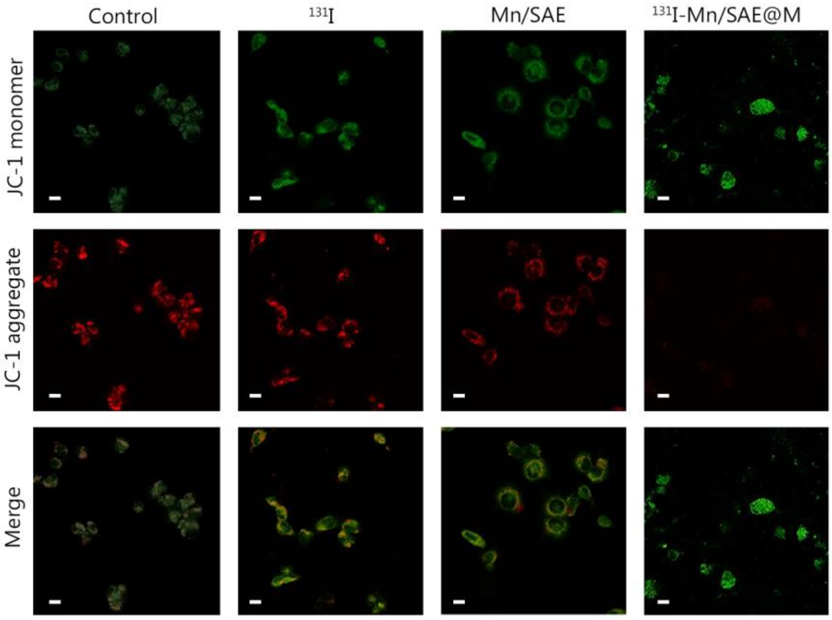

Fig. S18 Visualization of JC-1 monomer and JC-1 aggregate after different treatments. Scale bar = 10 μm. ^{131}I iodine-131, Mn/SAE manganese-based single-atom nanozyme, ^{131}I-Mn/SAE@M iodine-131-membrane-coated manganese single-atom nanozymes

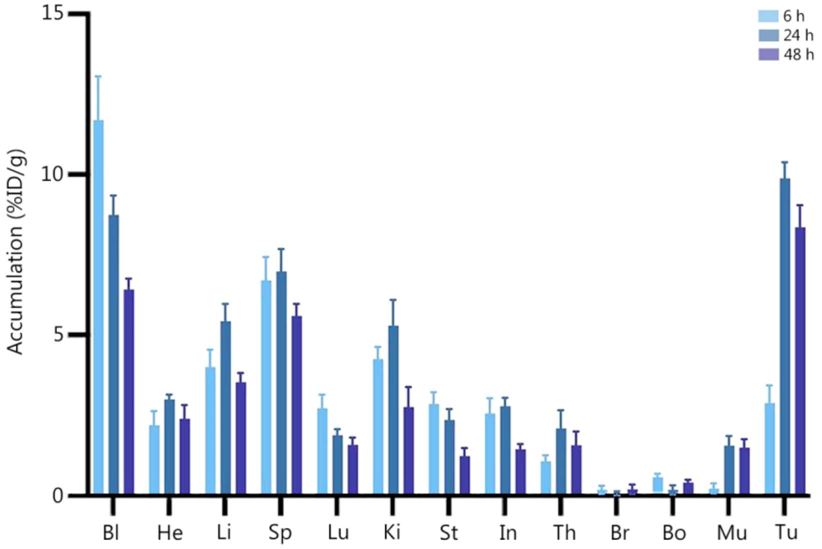

Fig. S19 Biodistribution of ^{131}I-Mn/SAE@M in LLC tumor-bearing mice at 6, 24, or 48 h post-intravenous injection. The three adjacent columns for each organ represent the results obtained at 6, 24, 48 h post-intravenous injection, respectively. Data are expressed as mean ± SD (*n* = 3). ^{131}I-Mn/SAE@M iodine-131-membrane-coated manganese single-atom nanozymes, SD standard deviation, LLC Lewis lung carcinoma, Bl blood, He heart, Li liver, Sp spleen, Lu lung, Ki kidney, St stomach, In intestines, Th thyroid, Br brain, Bo bone, M muscle, Tu tumor

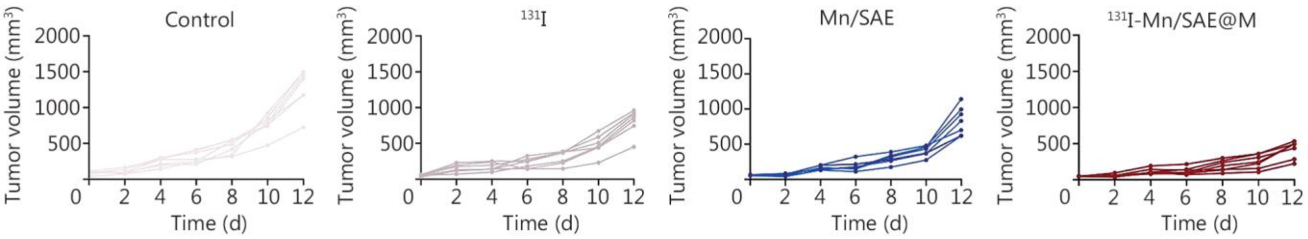

Fig. S20 Individual tumor growth curves of LLC tumor-bearing mice in corresponding treatment groups (*n* = 6). LLC Lewis lung carcinoma, ^{131}I iodine-131, Mn/SAE manganese-based single-atom nanozyme, ^{131}I-Mn/SAE@M iodine-131-membrane-coated manganese single-atom nanozymes

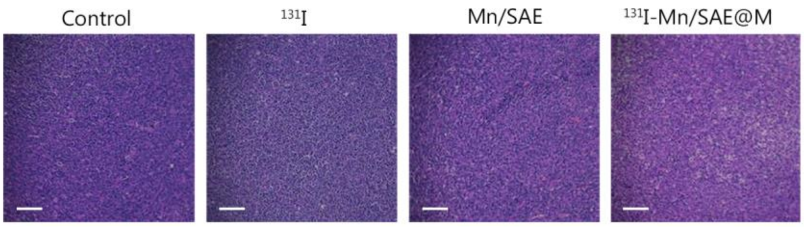

Fig. S21 Representative histological examinations of the tumor from each group with hematoxylin and eosin staining. Images of the main organs from mice injected with various treatment conditions. Scale bar = 100 μm. ^{131}I iodine-131, Mn/SAE manganese-based single-atom nanozyme, ^{131}I-Mn/SAE@M iodine-131-membrane-coated manganese single-atom nanozymes

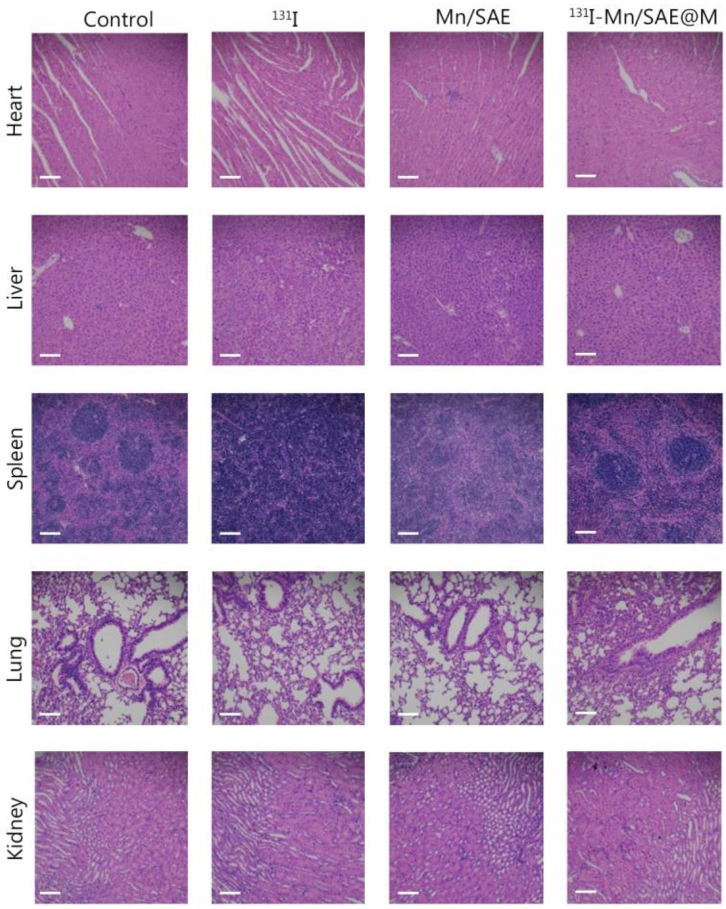

Fig. S22 Representative histological examinations of the main organs from each group with hematoxylin and eosin staining. Images of the main organs from mice injected with various treatment conditions. Scale bar = 100 μm. ^{131}I iodine-131, Mn/SAE manganese-based single-atom nanozyme, ^{131}I-Mn/SAE@M iodine-131-membrane-coated manganese single-atom nanozymes

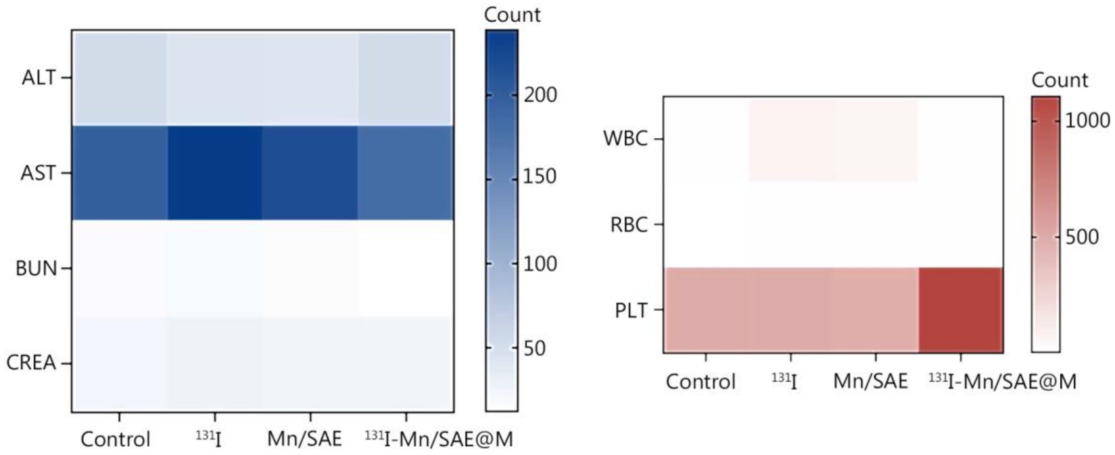

Fig. S23 Hemanalysis was performed on blood withdrawn from LLC tumor-bearing mice in corresponding treatment groups at the terminal of the study (n = 3). LLC Lewis lung carcinoma, ^{131}I iodine-131, Mn/SAE manganese-based single-atom nanozyme, ^{131}I-Mn/SAE@M iodine-131-membrane-coated manganese single-atom nanozymes, ALT alanine aminotransferase, AST aspartate aminotransferase, BUN blood urea nitrogen, CREA creatinine, WBC white blood cell, RBC red blood cell, PLT platelet

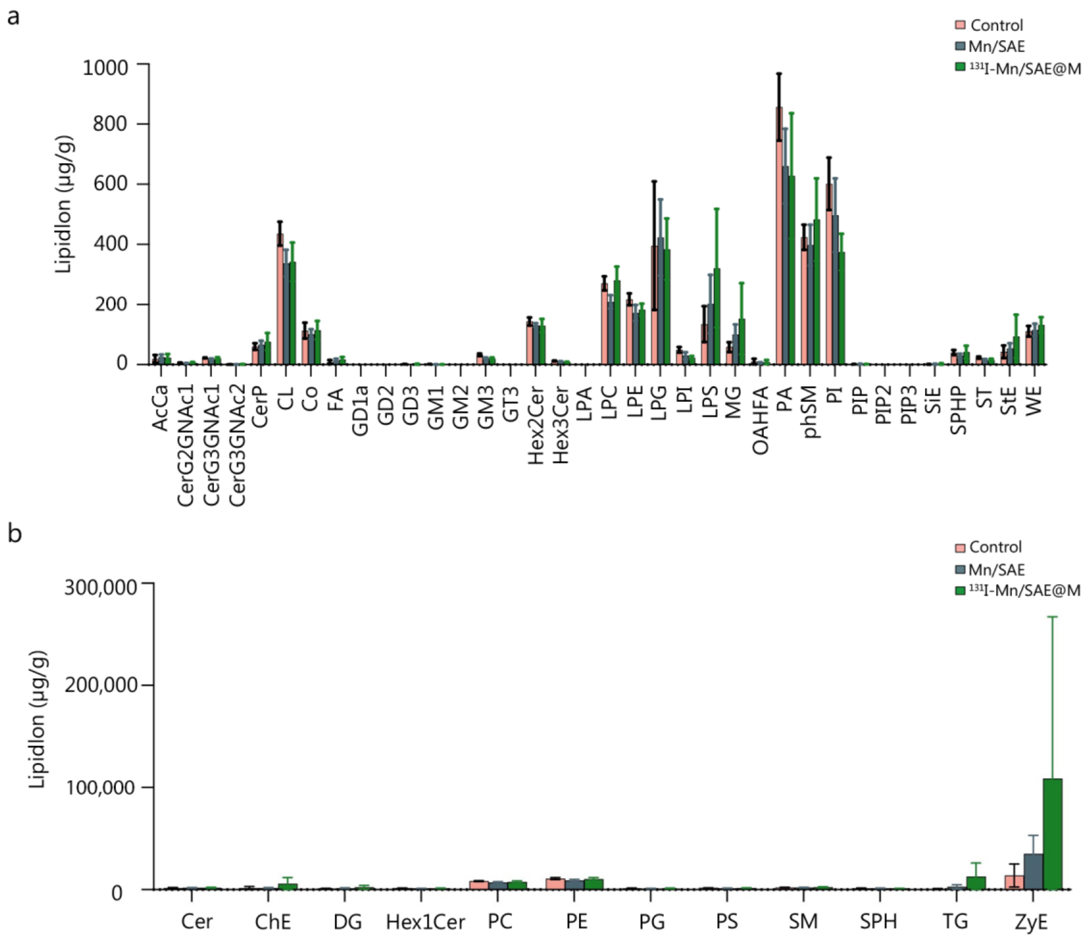

Fig. S24 Differences in the contents of each lipid subclass among different treatment groups. Data are expressed as mean ± SD (n = 3). Mn/SAE manganese-based single-atom nanozyme, ^{131}I-Mn/SAE@M iodine-131-membrane-coated manganese single-atom nanozymes, SD standard deviation AcCa acylcarnitine, CerG2GNAc1 dihexosylceramide with N-acetylglucosamine, CerG3GNAc1 trihexosylceramide with N-acetylglucosamine, CerG3GNAc2 trihexosylceramide with 2 N-acetylglucosamines, CerP ceramide-1-phosphate, CL cardiolipin, Co coenzyme, FA fatty acid, GD1a monosialotetrahexosylganglioside GD1a, GD1b monosialotetrahexosylganglioside GD1b, GD2 disialoganglioside GD2, GM1 monosialoganglioside GM1, GM2 monosialoganglioside GM2, GM3 monosialoganglioside GM3, GT3 trisialotetrahexosylganglioside GT3, Hex2Cer dihexosylceramide, Hex3Cer trihexosylceramide, LPA lysophosphatidic acid, LPC lysophosphatidylcholine, LPE lysophosphatidylethanolamine, LPG lysophosphatidylglycerol, LPI lysophosphatidylinositol, LPS lysophosphatidylserine, MG monoacylglycerol, OAHFA o-acyl-ω-hydroxy fatty acid, PA phosphatidic acid, PhSM phytosphingomyelin, PI phosphatidylinositol, PIP phosphatidylinositol monophosphate, PIP2 phosphatidylinositol bisphosphate, PIP3 phosphatidylinositol trisphosphate, S1P sphingosine-1-phosphate, SLE sulfo-lactosylceramide, SP sphingosine, ST sterol, SE sterol ester, WE wax ester, Cer ceramide,

ChE cholesteryl ester, DG diacylglycerol, Hex1Cer monohexosylceramide, PC phosphatidylcholine, PE phosphatidylethanolamine, PG phosphatidylglycerol, PS phosphatidylserine, SM sphingomyelin, SPH sphinganine, TG triacylglycerol, ZyE zymosterol ester

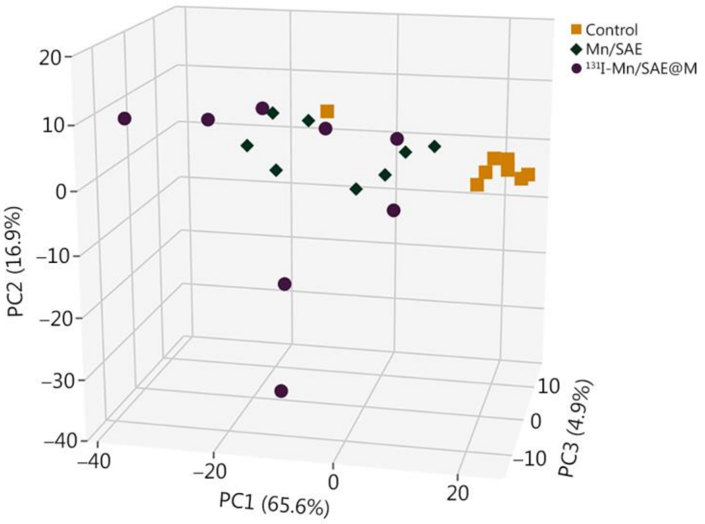

Fig. S25 Principal component analysis (PCA) score plot. PC1, PC2, and PC3 represent principal components 1, 2, and 3, respectively. Mn/SAE manganese-based single-atom nanozyme, ^{131}I-Mn/SAE@M iodine-131-membrane-coated manganese single-atom nanozymes

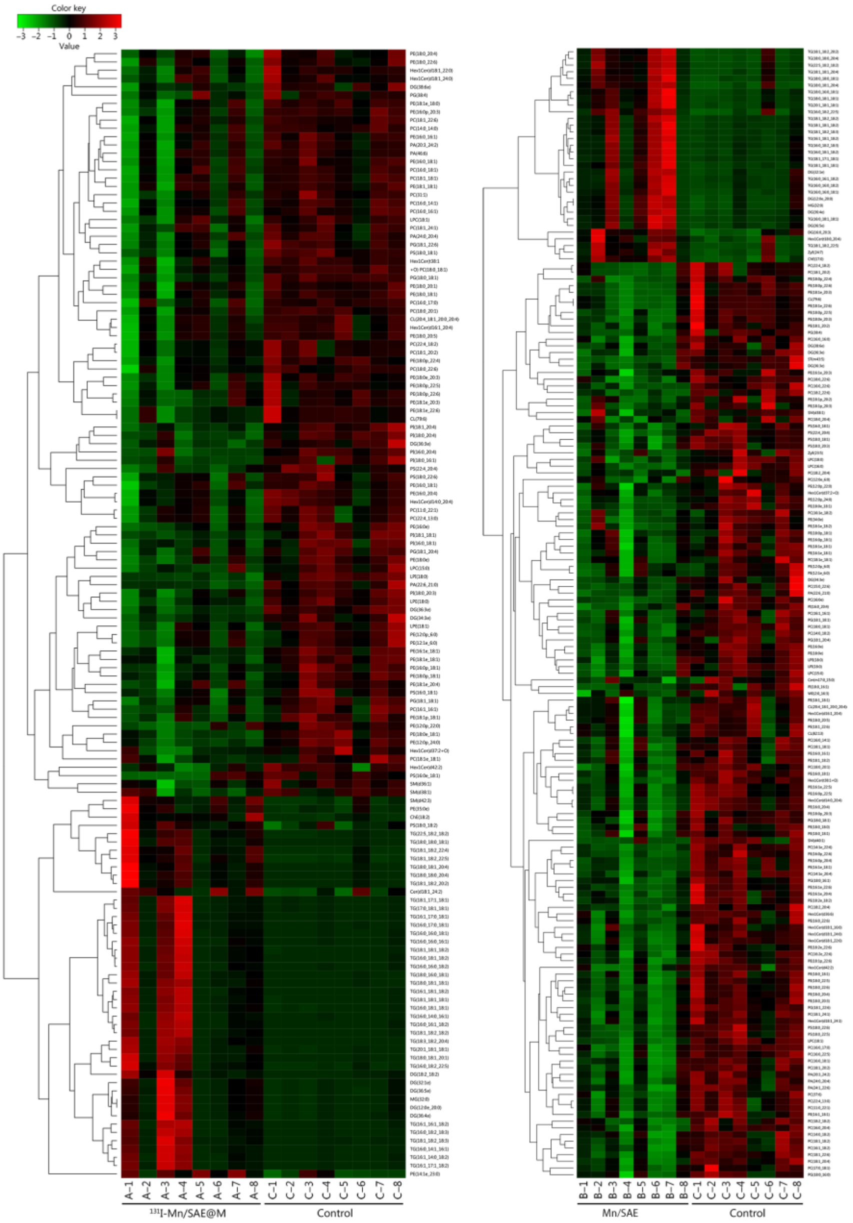

Fig. S26 Hierarchical clustering analysis based on significant difference in lipids. ^{31}I-Mn/SAE@M iodine-131-membrane-coated manganese single-atom nanozymes, Mn/SAE manganese-based single-atom nanozyme, PC phosphatidylcholine, PE phosphatidylethanolamine, PG phosphatidylglycerol, PI phosphatidylinositol, PS phosphatidylserine, PA phosphatidic acid, LPC lysophosphatidylcholine, LPE lysophosphatidylethanolamine, LPG lysophosphatidylglycerol, LPI lysophosphatidylinositol, LPS lysophosphatidylserine, LPA lysophosphatidic acid, Cer ceramide, Hex1Cer monohexosylceramide, Hex2Cer dihexosylceramide, Hex3Cer trihexosylceramide, SM sphingomyelin, dhCer dihydroceramide, dhSM dihydrosphingomyelin, CerP ceramide-1-phosphate, S1P sphingosine-1-phosphate, SPH sphingosine, MG monoacylglycerol, DG diacylglycerol, TG triacylglycerol, ChE cholesteryl ester, SE sterol ester, ST sterol, WE wax ester, ZgE zymosterol ester, GM1 monosialotetrahexosylganglioside GM1, GM2 monosialotetrahexosylganglioside GM2, GM3

monosialotetrahexosylganglioside GM3, GD1a disialoganglioside GD1a, GD1b disialoganglioside GD1b, GD2 disialoganglioside GD2, GT3 trisialotetrahexosylganglioside GT3, SLE sulfolactosylceramide, CL cardiolipin, Co coenzyme, FA fatty acid, OAHFA o-acyl-ω-hydroxy fatty acid, PhSM phytosphingomyelin

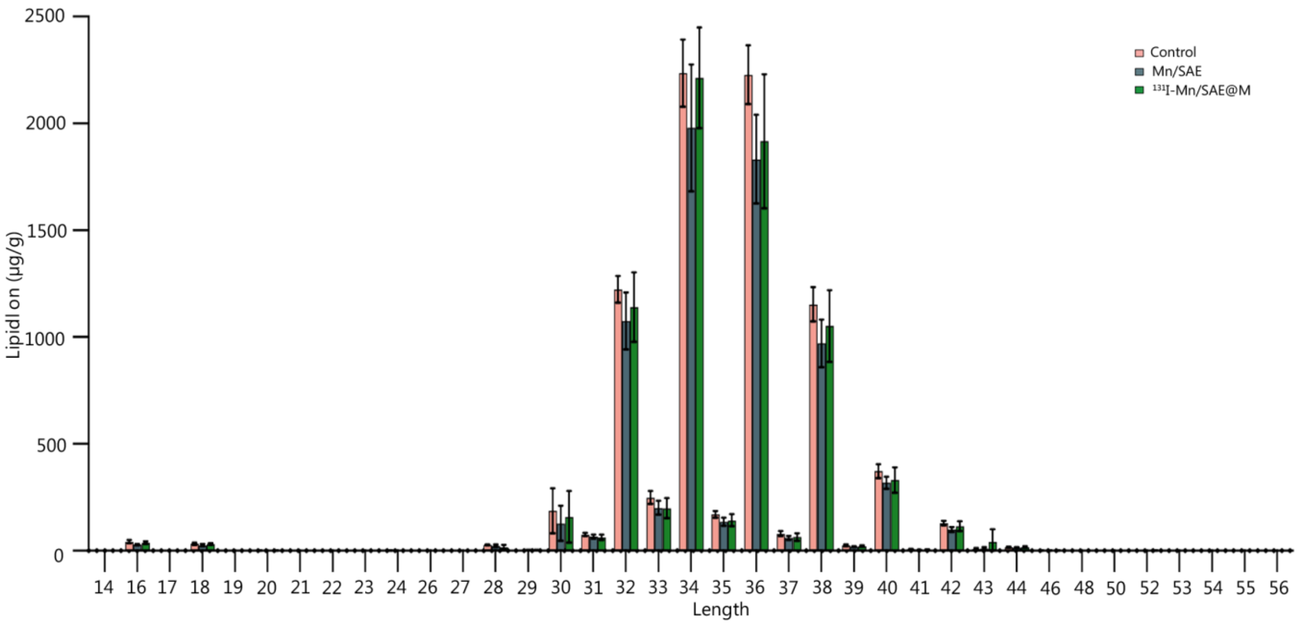

Fig. S27 Differences in the content of lipid molecules with various chain lengths under each class. Mn/SAE manganese-based single-atom nanozyme, ^{131}I-Mn/SAE@M iodine-131-membrane-coated manganese single-atom nanozymes

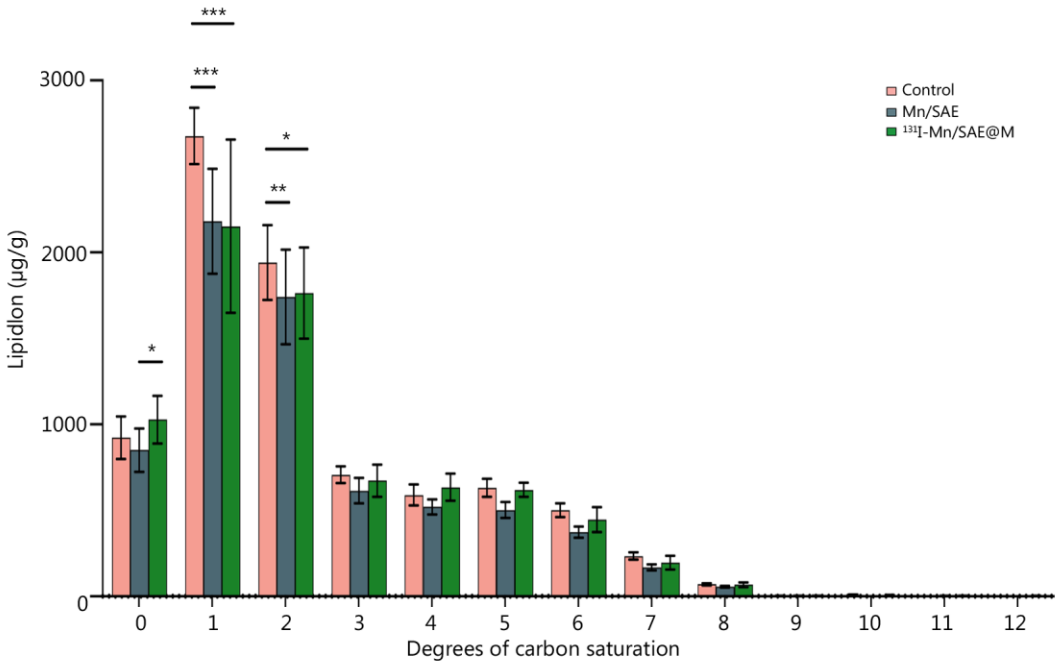

Fig. S28 Differences in the content of lipid molecules with various degrees of carbon saturation under each class using two-way ANOVA. Data are expressed as mean ± SD (n = 3). $^{*}P < 0.05$, $^{**}P < 0.01$, $^{***}P < 0.001$. SD standard deviation, Mn/SAE manganese-based single-atom nanozyme, ^{131}I-Mn/SAE@M iodine-131-membrane-coated manganese single-atom nanozymes

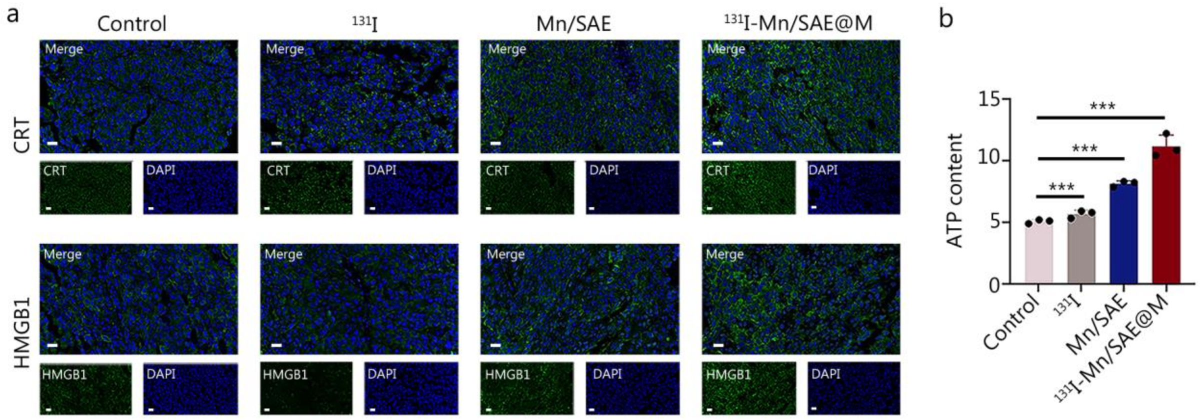

Fig. S29 Immunogenic cell death induced by ^{131}I-Mn/SAE@M. **a** Representative images of tumor tissues after CRT and HMGB1 immunofluorescence staining after different treatments as indicated. **b** The serum ATP contents after different treatments as indicated. Scale bar = 20 μm. Data are expressed as mean ± SD (n = 3). $^{***}P < 0.001$. ^{131}I-Mn/SAE@M iodine-131-membrane-coated manganese single-atom nanozymes, CRT calreticulin, HMGB1 high mobility group box 1, ATP adenosine triphosphate, SD standard deviation

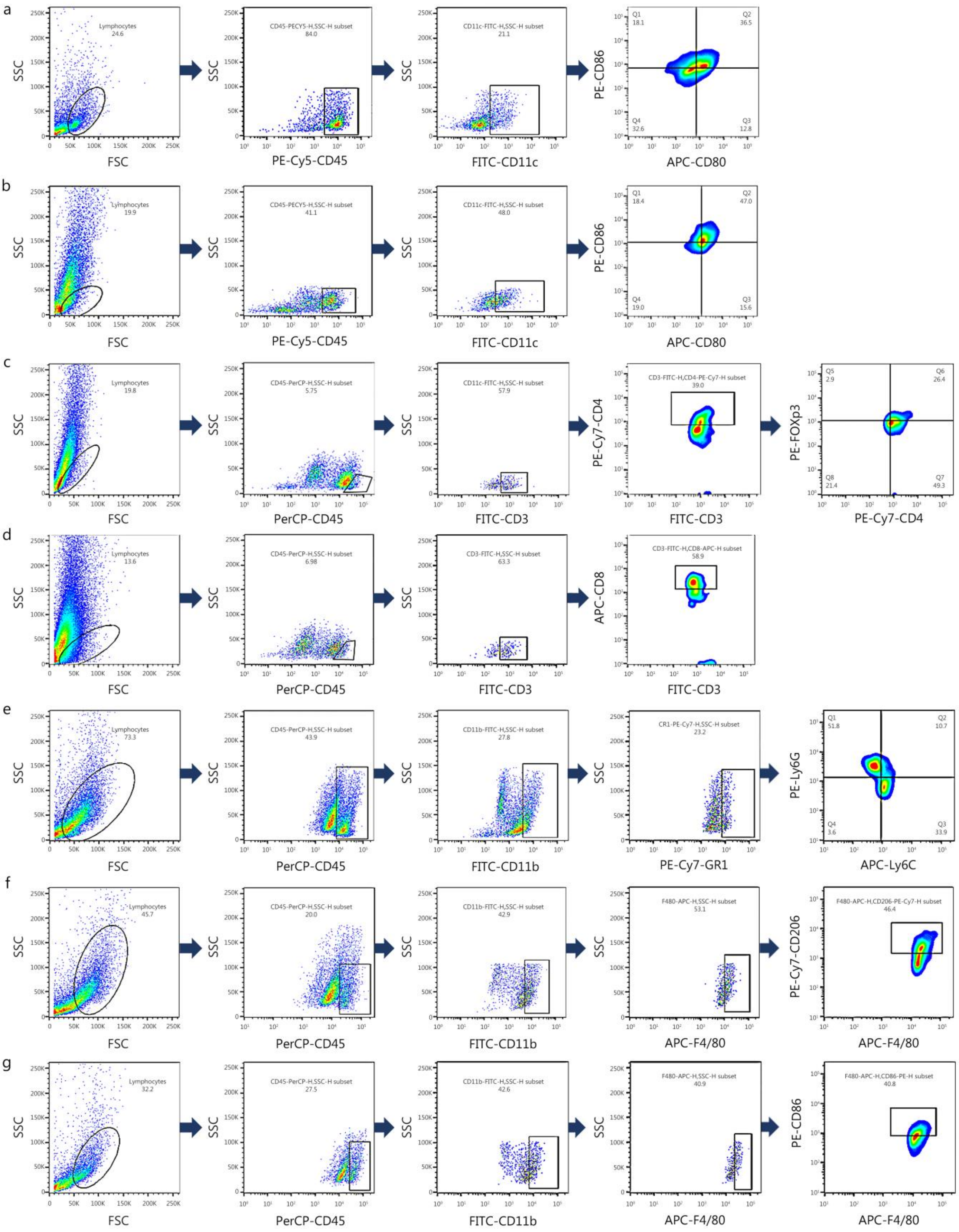

Fig. S30 Gating strategies. **a** Gating strategies for DCs (CD11c^{+}CD80^{+}CD86^{+}) in spleen presented on Fig. 6a. **b** Gating strategies for DCs (CD11c^{+}CD80^{+}CD86^{+}) in tumor presented on Fig. 6b. **c** Gating strategies for CD4^{+} T (CD3^{+}CD4^{+}) cells and Tregs (CD3^{+}CD4^{+}FoxP3^{+}) in tumor presented on Fig. 6c and e. **d** Gating strategies for CD8^{+} T (CD3^{+}CD8^{+}) cells in tumor presented on Fig. 6d. **e**

Gating strategies for MDSCs ($CD11b^+GR1^+Ly6G^+Ly6C^-$) in tumor presented on Fig. 6f. **f** Gating strategies for M2 phenotype TAMs ($F4/80^+CD206^+$) in tumors presented on Fig. 6g. **g** Gating strategies for M1 phenotype TAMs ($F4/80^+CD86^+$) in tumors presented on Fig. 6h. DCs dendritic cells, MDSCs marrow-derived suppression cells, TAMs tumor-associated macrophages, Tregs regulatory T cells, SSC side scatter, FSC forward scatter, FITC fluorescein isothiocyanate, PE-Cy5 phycoerythrin-cyanine 5, APC allophycocyanin, FOXp3 forkhead box p3

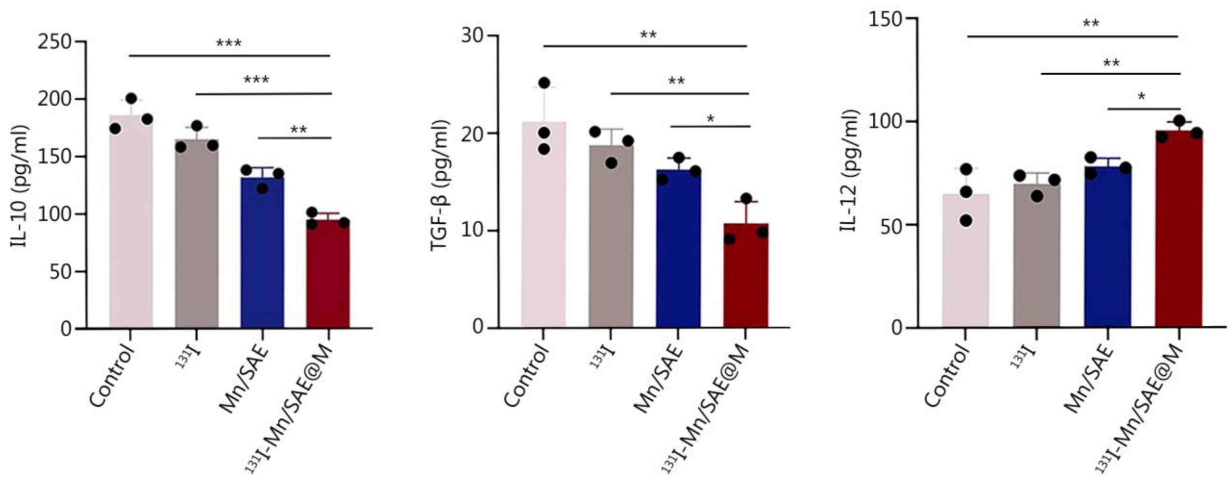

Fig. S31 Expression levels of serum cytokines, including IL-10, TGF-β, and IL-12 in the mice after different treatments. Data are expressed as mean ± SD (n = 3). $^{*}P < 0.01$, $^{**}P < 0.001$. SD standard deviation, TGF-β transforming growth factor-β, IL-10 interleukin-10, IL-12 interleukin-12, ^{131}I iodine-131, Mn/SAE manganese-based single-atom nanozyme, ^{131}I-Mn/SAE@M iodine-131-membrane-coated manganese single-atom nanozymes

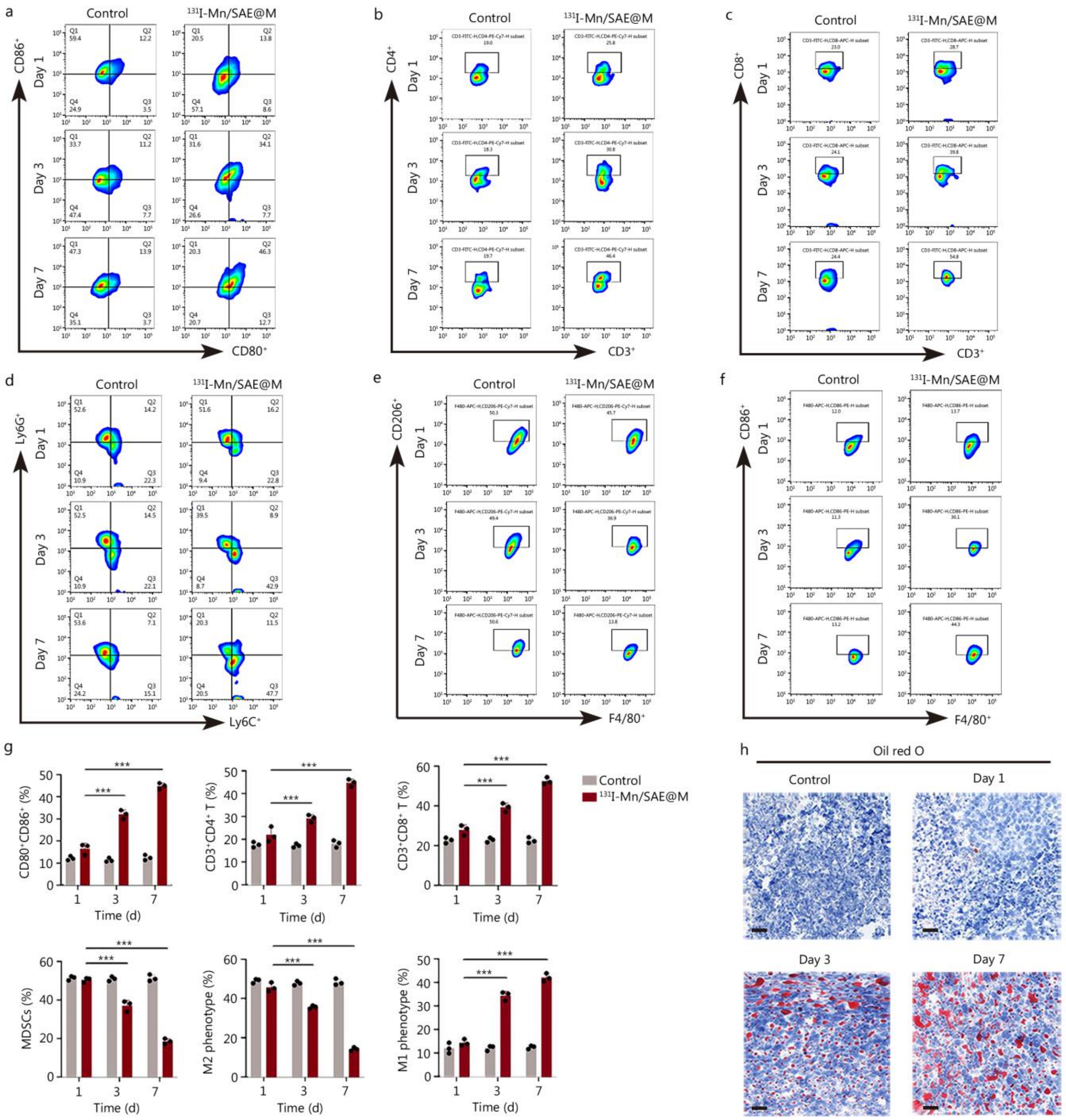

Fig. S32 Immune response activation and lipid metabolism disruption after treatment with ^{131}I-Mn/SAE@M. **a** Representative flow cytometry plots showing DC maturation in tumors. **b** Representative flow cytometry plots showing $CD4^+$ T cells in tumors. **c** Representative flow cytometry plots showing $CD8^+$ T cells in tumors. **d** Representative flow cytometry plots showing MDSCs in tumors. **e** Representative flow cytometry plots showing M2 phenotype TAMs in tumors. **f** Representative flow cytometry plots showing M1 phenotype TAMs in tumors. **g** The statistical data (n = 3 per group). **h** Representative optical microscopy images of lipid droplets (red) in tumor sections after different treatments with Oil red O staining. Data are expressed as mean ± SD (n = 3). Scale bar = 20 μm. $^{***}P < 0.001$. SD standard deviation, DCs dendritic cells, MDSCs marrow-derived

suppression cells, TAMs tumor-associated macrophages, ^{131}I-Mn/SAE@M iodine-131-membrane-coated manganese single-atom nanozymes

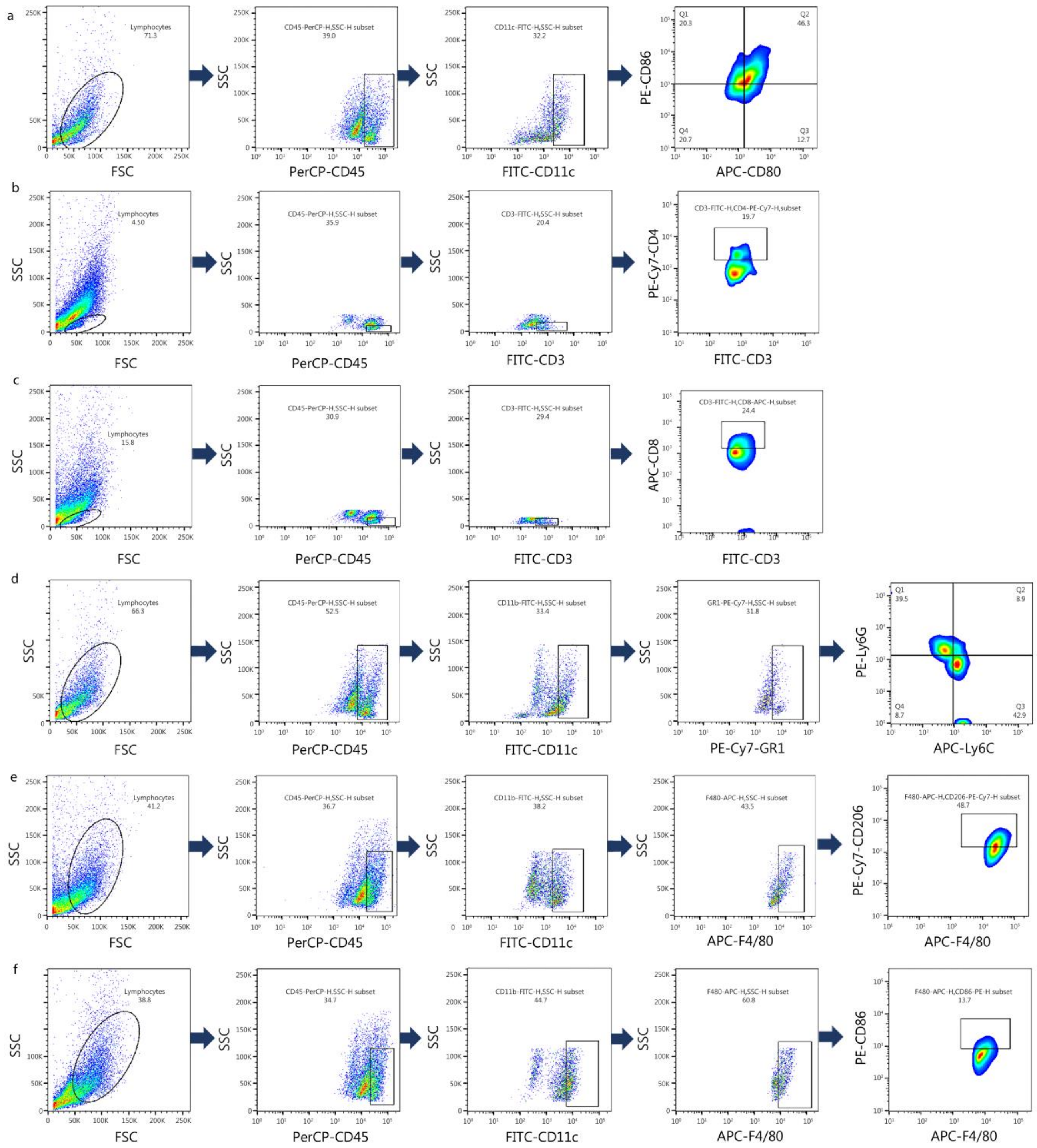

Fig. S33 Gating strategies. **a** Gating strategies for DCs (CD11c$^+$CD80$^+$CD86$^+$) in tumor presented on Fig. S32a. **b** Gating strategies for CD4$^+$ T (CD3$^+$CD4$^+$) cells in tumor presented on Fig. S32b. **c** Gating strategies for CD8$^+$ T (CD3$^+$CD8$^+$) cells in tumor presented on Fig. S32c. **d** Gating strategies for MDSCs (CD11b$^+$GR1$^+$Ly6G$^+$Ly6C$^-$) in tumor presented on Fig. S32d. **e** Gating strategies for M2 phenotype TAMs (F4/80$^+$CD206$^+$) in tumors presented on Fig. S32e. **f** Gating strategies for M1 phenotype TAMs (F4/80$^+$CD86$^+$) in tumors presented on Fig. S32f. DCs dendritic cells, MDSCs marrow-derived suppression cells, TAMs tumor-associated macrophages, SSC side scatter, FSC

forward scatter, FITC fluorescein isothiocyanate, PE-Cy5 phycoerythrin-cyanine 5, APC allophycocyanin

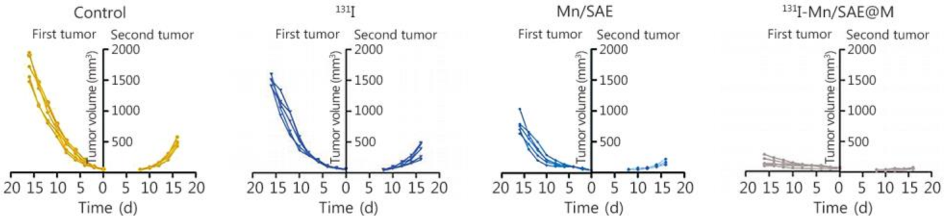

Fig. S34 Individual tumor growth curves of LLC tumor-bearing mice in the corresponding treatment groups (*n* = 5). LLC Lewis lung carcinoma, ^{131}I iodine-131, Mn/SAE manganese-based single-atom nanozyme, ^{131}I-Mn/SAE@M iodine-131-membrane-coated manganese single-atom nanozymes

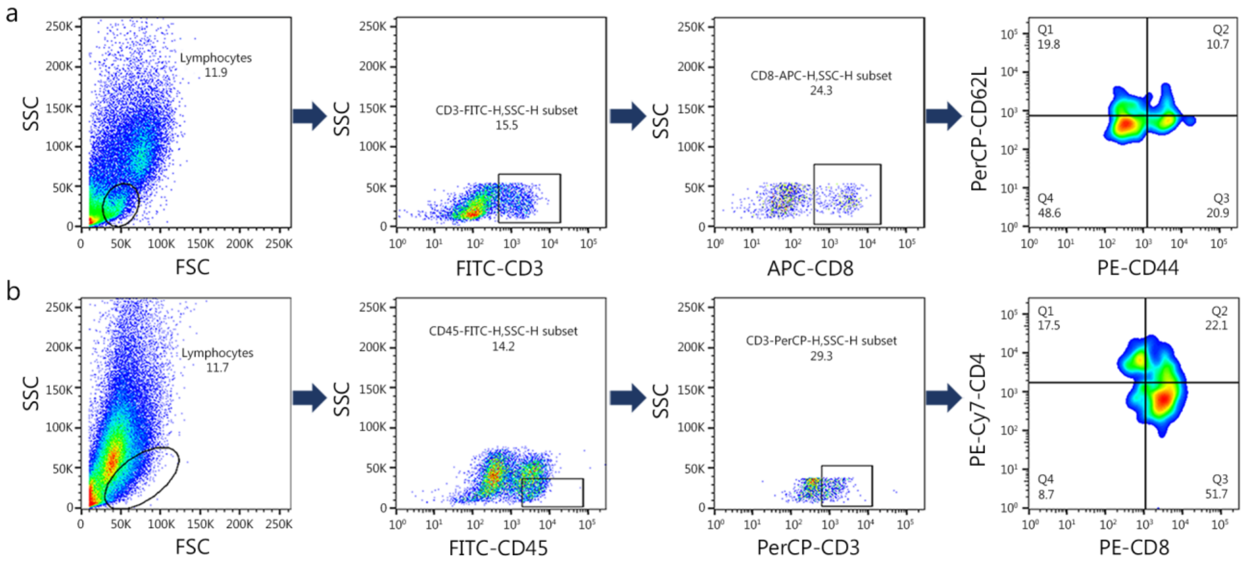

Fig. S35 Gating strategies. **a** Gating strategies for immune effector memory T cells (T_{EM}: $CD3^+CD8^+CD44^+CD62L^-$) in spleen presented on Fig. 7e. **b** Gating strategies for $CD8^+$ T cells ($CD3^+CD8^+$) in distant tumor presented on Fig. 7f. SSC side scatter, FSC forward scatter, FITC fluorescein isothiocyanate, APC allophycocyanin, PE phycoerythrin, PerCP peridinin-chlorophyll protein
